# Anterior Cingulate Cortex Signals the Need to Control Intrusive Thoughts during Motivated Forgetting

**DOI:** 10.1523/JNEUROSCI.1711-21.2022

**Published:** 2022-05-25

**Authors:** Maité Crespo-García, Yulin Wang, Mojun Jiang, Michael C. Anderson, Xu Lei

**Affiliations:** ^1^MRC Cognition and Brain Sciences Unit, University of Cambridge, Cambridge CB2 7EF, United Kingdom; ^2^Sleep and NeuroImaging Center, Faculty of Psychology, Southwest University, Chongqing 400715, People's Republic of China; ^3^Key Laboratory of Cognition and Personality, Ministry of Education, Chongqing 400715, People's Republic of China; ^4^Behavioural and Clinical Neurosciences Institute, University of Cambridge, Cambridge CB2 3EB, United Kingdom

**Keywords:** anterior cingulate cortex, dorsolateral prefrontal cortex, inhibitory control, intrusive thoughts, memory suppression, motivated forgetting

## Abstract

How do people limit awareness of unwanted memories? When such memories intrude, a control process engages the right DLPFC (rDLPFC) to inhibit hippocampal activity and stop retrieval. It remains unknown how the need for control is detected, and whether control operates proactively to prevent unwelcome memories from being retrieved, or responds reactively, to counteract intrusions. We hypothesized that dorsal ACC (dACC) detects the emergence of an unwanted trace in awareness and transmits the need for inhibitory control to rDLPFC. During a memory suppression task, we measured in humans (both sexes) trial-by-trial variations in the theta power and N2 amplitude of dACC, two EEG markers that are thought to reflect the need for control. With simultaneous EEG-fMRI recordings, we tracked interactions among dACC, rDLPFC, and hippocampus during suppression. We found a clear role of dACC in detecting the need for memory control and upregulating prefrontal inhibition. Importantly, we identified distinct early (300–450 ms) and late (500–700 ms) dACC contributions, suggesting both proactive control before recollection and reactive control in response to intrusions. Stronger early activity was associated with reduced hippocampal activity and diminished BOLD signal in dACC and rDLPFC, suggesting that preempting retrieval reduced overall control demands. In the later window, dACC activity was larger, and effective connectivity analyses revealed robust communication from dACC to rDLPFC and from rDLPFC to hippocampus, which are tied to successful forgetting. Together, our findings support a model in which dACC detects the emergence of unwanted content, triggering top-down inhibitory control, and in which rDLPFC countermands intruding thoughts that penetrate awareness.

**SIGNIFICANCE STATEMENT** Preventing unwanted memories from coming to mind is an adaptive ability of humans. This ability relies on inhibitory control processes in the prefrontal cortex to modulate hippocampal retrieval processes. How and when reminders to unwelcome memories come to trigger prefrontal control mechanisms remains unknown. Here we acquired neuroimaging data with both high spatial and temporal resolution as participants suppressed specific memories. We found that the anterior cingulate cortex detects the need for memory control, responding both proactively to early warning signals about unwelcome content and reactively to intrusive thoughts themselves. When unwanted traces emerge in awareness, anterior cingulate communicates with prefrontal cortex and triggers top-down inhibitory control over the hippocampus through specific neural oscillatory networks.

## Introduction

Suppressing unwanted memories engages the dorsal anterior cingulate cortex (dACC), but its contribution to inhibitory control over memory remains unclear. In nonmemory contexts, major theoretical accounts agree that dACC monitors ongoing processing and detects information indicating a need to intensify cognitive control, and that dACC communicates this demand to prefrontal regions that implement control (Botvinick et al., 2001; Botvinick, 2007; Alexander and Brown, 2011; Cavanagh and Frank, 2014; Alexander and Brown, 2015; Vassena et al., 2020). The conflict monitoring theory (Botvinick et al., 2001) proposes that dACC is sensitive to cognitive conflict, and that processed conflict signals initiate strategic adjustments in cognitive control to prevent future conflict. Accounts derived from the predicted response outcome model (Alexander and Brown, 2011) point out that surprising events typically increase the activation of this region, so they maintain that dACC plays a specific role in calculating surprise (Vassena et al., 2020). Following these ideas, we hypothesized that, during motivated forgetting, dACC dynamically regulates mnemonic inhibition by computing signals that indicate a need to control unwelcome content. On one hand, these warning signals may originate from reminders that foreshadow the intrusion of an unwanted memory, triggering proactive control to prevent retrieval; on the other hand, they may derive directly from the reactivation of an unwanted memory, which may elicit cognitive conflict and a need to purge the intruding memory from mind (Levy and Anderson, 2012). Specifically, when proactive control fails to prevent retrieval, intrusion-related activity would drive stronger signals in dACC as the demands for cognitive control increase, and this would initiate a reactive mechanism engaging right dorsolateral prefrontal cortex (rDLPFC) to downregulate the hippocampus (Levy and Anderson, 2012; Benoit et al., 2015; Gagnepain et al., 2017). We hypothesized that dACC transmits these proactive and reactive signals to rDLPFC to amplify top-down inhibition over regions driving retrieval of the offending memory.

**Figure 1.**
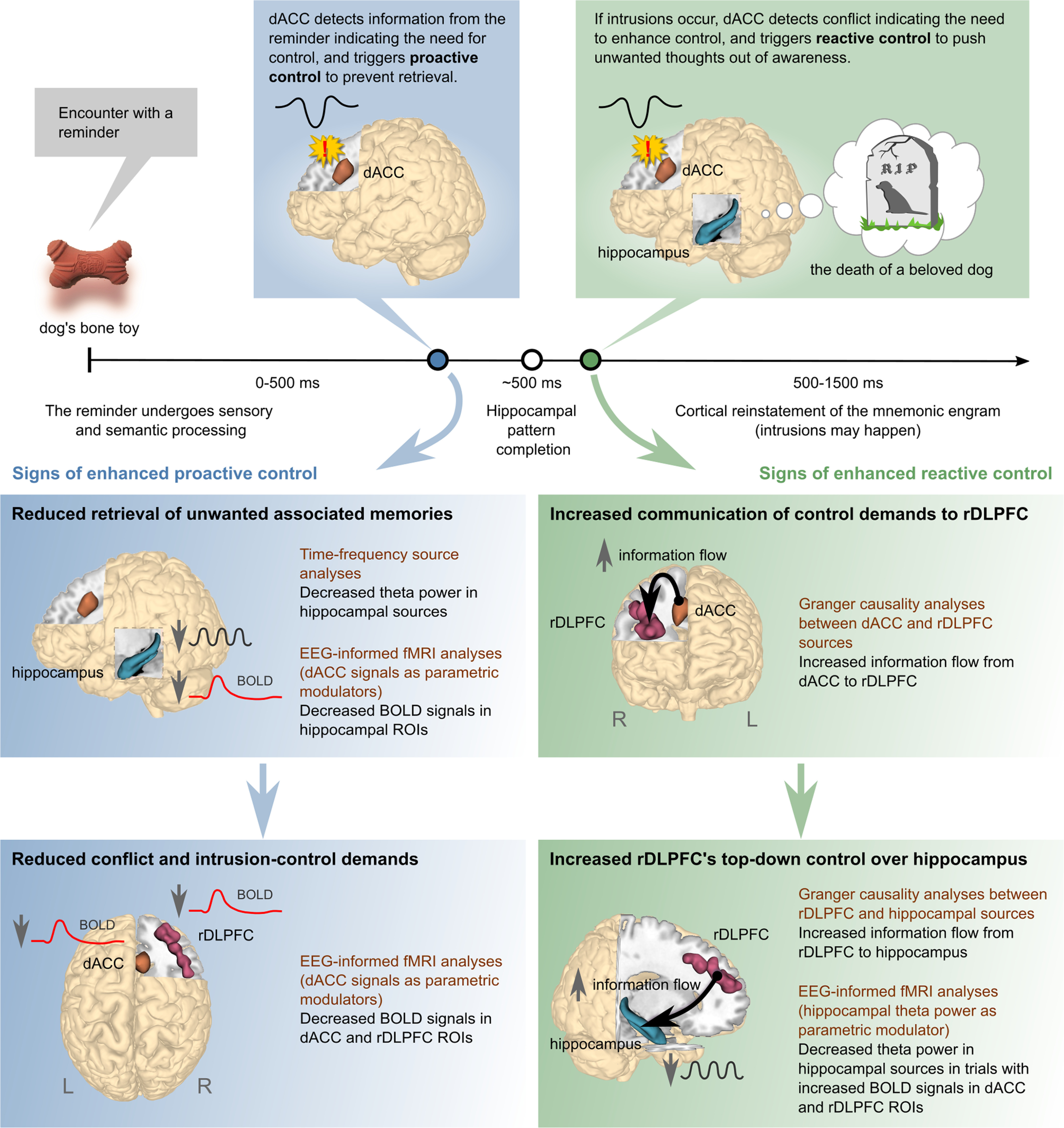
Summary of expected relationships between EEG measures and BOLD signals associated with proactive and reactive control. Top, A hypothetical timeline of brain processes after encountering a reminder (a dog's bone toy) associated with an unwanted memory (the death of a beloved dog). Proactive control (blue dot) is triggered before episodic retrieval of associated memories starts in the hippocampus, whereas reactive control (green dot) is triggered after, because of conflict generated by intrusions. Bottom panels summarize the expected effects of enhanced proactive (blue boxes) and reactive (green boxes) control, according to the model. These effects imply specific relationships between EEG measures and BOLD signals, which were tested using the methods listed (brown text). Please refer to the main text for more details. R, Right brain hemisphere; L, Left brain hemisphere.

To test these hypotheses, we acquired simultaneous EEG-fMRI recordings as participants performed a memory suppression task. This multimodal approach allowed us to relate temporally precise EEG signatures of the need for enhanced cognitive control to anatomically precise BOLD signals to track dynamic interactions among the dACC, rDLPFC, and hippocampus during suppression (Fig. 1). The need for cognitive control (whether proactive or reactive) was indexed by transient increases in frontal-midline theta power, following a well supported hypothesis about the functional role of this EEG marker (Cavanagh et al., 2012; Cavanagh and Frank, 2014; Cavanagh and Shackman, 2015). Using an EEG-informed fMRI approach, we first tested, on a trial-by-trial basis, the coupling between BOLD signals in the foregoing regions and early EEG effects that might reflect proactive control. We indexed putative proactive control via measures of frontal-midline theta power and N2 amplitude arising before 500 ms, before the likely recollection of the intruding memory. We reasoned that because intrusions are experienced in conscious awareness, intrusion-driven reactive control could only happen after hippocampal pattern completion (∼500 ms) and within the time window wherein cortical reinstatement could occur (500–1500 ms; Staresina and Wimber, 2019). We hypothesized that this early proactive control prepares the system to inhibit hippocampal retrieval. On the other hand, trials with poorer proactive control would lead to increased demands for intrusion control later in the trial, reactively triggering elevated activity in both dACC and rDLPFC (Fig. 1, blue boxes). To tie such delayed prefrontal activations to reactive control, we examined how dACC signaling determined both communication between dACC and rDLPFC and the suppression of putative retrieval processes indexed by hippocampal theta oscillations. To achieve this, we used temporally resolved EEG source analyses to investigate regional modulations that likely occurred after recollection onset (∼500 ms onward). Then, we captured the dynamics of information flow during reactive control of unwanted memories by calculating Granger causality (GC) between EEG sources. These analyses allowed us to measure whether dACC transmits control signals to rDLPFC, and whether rDLPFC, in turn, intensifies top-down inhibition of the hippocampus, facilitating motivated forgetting (Fig. 1, green boxes).

## Materials and Methods

### Participants

A total of 24 participants (12 females; mean ± SD age, 21.4 ± 2.0 years) were recruited through the Southwest University undergraduate participant pool. All had normal or corrected-to-normal vision and had no history of psychiatric or neurologic illness. To check whether the participants had adequate sleep before the experiment, they answered questions about their sleep state on arrival at the laboratory; all of them were in line with our requirements. None of the participants had experienced the experimental task before. The Ethics Committee of Southwest University approved the study. Written informed consent was obtained from all participants according to the Declaration of Helsinki after detailed explanation of the experiment protocol. All of the participants received monetary compensation after their participation.

### Stimuli

One hundred twenty-eight neutral words were selected from the *Thesaurus of Modern Chinese* to form 64 pairs of weakly related words. Within each pair, a word was used as a cue and the other word as an associate. Each associate word was a member of a unique semantic category, so it could be later recalled using that extra-list category name as cue. Forty-eight word pairs were divided into three sets of 16 word pairs, which rotated across participants through the conditions [Think (T), No-Think (NT), and Baseline (B)]. The remaining 16 pairs were used as fillers for practice. Eight additional single words were included during the Think/No-Think task (TNT) task in a perceptual baseline condition.

### Experimental design and statistical analyses

#### Procedure.

**Figure 2.**
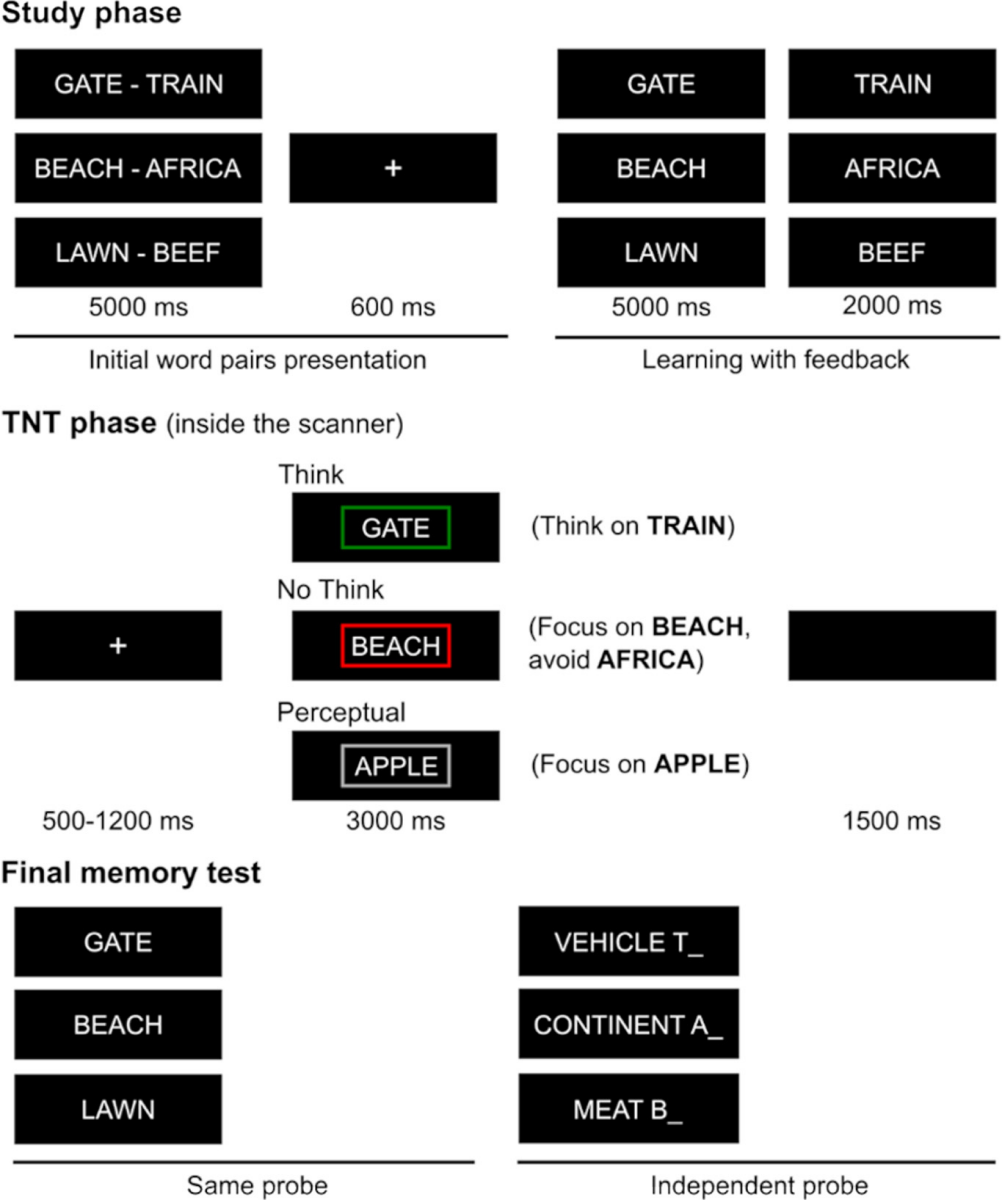
Experimental paradigm. In the study phase, participants encoded cue–associate word pairs. During the scanned TNT phase, participants recalled some of the associates (Think, cues presented inside a green frame) and suppressed others (No-Think, cues presented inside a red frame). Participants paid full attention to unpaired words (without associate) presented inside a gray frame (Perceptual). In the final memory phase, participants were asked to recall the associates given their cues (same probe) or their category name (independent probe). Memory was also evaluated for some word pairs that were initially learned but did not enter the TNT phase (Baseline).

The experiment consisted of the following three phases: study, TNT, and final memory test (Fig. 2).

#### Study phase.

First, participants studied all the 64 cue–associate word pairs. On each trial, both words were displayed visually, side by side, on a black background for 5 s. Each trial was separated by an interstimulus interval (ISI) with a fixation cross for 600 ms. Then, participants were trained to recall the associate words given the cues. On each trial, a cue appeared at the center of the screen for 5 s, and participants were asked to recall and say out loud the corresponding associate word. Participants' responses were recorded. After every trial, the associate word was displayed as feedback for 2 s. All word pairs were repeatedly trained until participants correctly provided at least 50% of all the associate words. Finally, participants were tested again by displaying each cue, but the associate feedback was omitted. This test was used to identify the word pairs that participants successfully learned before entering the TNT phase and restrict (conditionalize) the analyses to those trials corresponding to learned associations.

#### TNT phase.

Participants performed this part of the experiment inside the fMRI scanner, and stimuli were displayed on a back-projection screen mounted above participants' heads. At the beginning of this phase, participants practiced the task on 16 fillers. Afterward, short diagnostic questionnaires were administered to assess whether participants understood the instructions, and questions were clarified. The proper task was divided into six blocks separated by 1 min breaks. Each block consisted of 80 trials, where all cues from the Think (16) and No-Think (16) conditions, together with Perceptual baseline words (8), were presented twice. In sum, each cue word was presented 12 times during this phase. Each trial started with a fixation cross (variable ISI between 500 and 1200 ms). Then, a cue word appeared within a colored frame for 3 s. The trial ended with a blank screen (ISI = 1.5 s). For cues within green frames (Think), participants were asked to think of the associate word and keep it in mind while the cue was on the screen. For cues within red frames (No-Think), participants were asked to pay full attention to the cue and prevent the associate word from coming into mind during the whole trial. Instructions encouraged that participants followed a direct suppression strategy (Bergström et al., 2009; Benoit and Anderson, 2012), by emphasizing that they should suppress retrieval and avoid replacing the associate with alternative words or thoughts. For words within a gray frame, participants were just asked to pay attention to them.

#### Final memory test.

Memory for all studied word pairs was evaluated, including Baseline items that were excluded from the TNT phase. Until this phase, participants were unaware of a final memory assessment to prevent the influence of anticipatory mechanisms on forgetting scores and were initially told that the experiment was about attention and their ability to ignore distraction. Participants performed the following two types of final tests: same probe (SP) and independent probe (IP), with the order of these tests counterbalanced across participants. On the SP test, each cue was presented again, and participants were asked to recall and say out loud the associate word, as they did during the training phase. On the IP test, each unique category name was given as the cue, and participants were asked to recall and say out loud a member of this category, from those associate words they initially studied. At the end of the experiment, participants completed a questionnaire to determine whether they followed the instructions to suppress retrieval during No-Think trials.

#### EEG.

EEG data were recorded by 32 Ag/Cl electrodes that were placed on the scalp according to the international 10/20 system. The data were digitized at 5 kHz, referenced online to FCz using a nonmagnetic MRI-compatible EEG system (BrainAmp MR Plus, Brain Products). Impedances were kept to <10 kΩ before recording. Electrocardiogram was simultaneously acquired from each participant. The EEG amplifier used a rechargeable power pack that was placed outside the scanner bore. To ensure the temporal stability of the EEG acquisition in relation to the switching of the gradients during the MR acquisition, a SyncBox (SyncBox MainUnit, Brain Products) was used to synchronize the amplifier system with the MRI scanner system. Fiber-optic cables transmitted the amplified and digitized EEG signal to the recording computer, which was outside the scanner room. An adapter (BrainAmp USB-Adapter, Brain Products) was used to convert the optical signal into the electrical signal.

#### fMRI.

All images were acquired using a 3 T Trio scanner (Siemens). A T2-weighted gradient EPI sequence (TR = 1500 ms; TE = 29 ms; FOV = 192 × 192 mm^2^; flip angle = 90°; acquisition matrix = 64 × 64; thickness, 5 mm; gap = 0.5 mm; in-plane resolution = 3.0 × 3.0 mm^2^; axial slices = 25) was used for functional image acquisition. The first three volumes of each sequence were discarded to account for magnetization saturation effects. All subjects were scanned in six blocks, where each block lasted 435 s and contained 290 volumes. After the first three blocks, a T1 scan was acquired for 5 min, where participants were told to relax and hold still. The 3D spoiled gradient recalled sequence used the following parameters: TR = 8.5 ms; TE = 3.4 ms; FOV = 240 × 240 mm^2^; flip angle = 12°; acquisition matrix = 512 × 512; thickness = 1 mm with no gap. The high-resolution T1-weighted structural volume provided an anatomic reference for the functional scan. We minimized head movements by using a cushioned head fixation device.

#### Behavioral data analysis.

Recall accuracies at the final memory test were estimated for Think, No-Think, and Baseline conditions, and for each test type (SP and IP) separately. The analyses were conditionalized: only word pairs learned in the study phase (determined by the memory test prior the TNT phase) were considered. Recall accuracies were computed as a ratio between the number of word pairs correctly recalled at the final test and the total number of word pairs that were learned at study. These measures were compared using a two-way ANOVA with the memory condition (No-Think and Baseline) and test type (SP and IP) as factors, to determine whether there was below-baseline forgetting. Paired-samples *t* tests were applied to assess the effect of memory suppression and memory retrieval on final recall performance within each test type. We conducted all behavioral statistical analyses in SPSS Statistics version 19.0.

#### EEG data preprocessing.

Main fMRI gradient and ballistocardiogram (BCG) artifacts were first removed from the EEG data acquired during the TNT phase, following standard template subtraction procedures. Subsequently, data were down-sampled to 250 Hz and digitally filtered within 0.1–45 Hz using a Chebyshev II-type filter. Temporal independent component analysis (ICA; (Bell and Sejnowski, 1995)) was subsequently applied to attenuate ocular artifacts (e.g., blinks, saccades), frontotemporal muscular activity of small intensity and residual BCG and imaging artifacts. Artifactual components were visually selected on the base of their characteristic time courses, topographic amplitude distributions, signal features (i.e., kurtosis, energy), and spectral characteristics (Mayeli et al., 2016). Continuous EEG data were divided into 5000 ms segments relative to the onset of all words presented during the TNT phase (Think, No-Think, and Perceptual Baseline). Each segment included 500 ms of prestimulus baseline and 4500 ms of poststimulus period. Segments that were contaminated by jumps, movement, or strong muscular activity were removed. Finally, EEG signals were rereferenced to the average for further analyses. All of the following analyses were conditionalized as the behavioral measures by including only trials belonging to word pairs that were learned at study.

#### Event-related potential analyses.

Event-related potentials (ERPs) were computed within the 1250 ms epoch comprising 250 ms of prestimulus baseline and 1000 ms of poststimulus period. The suppression-N2 component was identified by visual inspection of the grand-mean ERPs of frontocentral sensors, around the latencies reported in previous studies (Bergström et al., 2009; Mecklinger et al., 2009; Waldhauser et al., 2012; Streb et al., 2016). We focused on the suppression-N2 effect between 350 and 450 ms, which has been shown to correlate with the traditional motor N2 effect in the stop signal task in the same participants (Mecklinger et al., 2009) and with suppression-induced forgetting (SIF; Streb et al., 2016). Other studies have reported suppression-N2 effects starting from 300 ms (Bergström et al., 2009; Waldhauser et al., 2012); therefore, statistical comparisons between No-Think and Think N2 waveforms were limited to the 300–450 ms time window. Note that early studies have also found N2-like effects at earlier latencies [at 180–225 ms in the early negativity (Bergström et al., 2009); and at 200–300 ms (Bergström et al., 2007)] during memory suppression, but these effects have been found with specific methodological manipulations that we did not use here. We did not include these earlier latencies in our N2 analyses because we did not find amplitude differences in the frontocentral channel after visual inspection or in control statistical analyses. A first analysis contrasted the mean amplitudes of all sensors using nonparametric two-sample paired *t* tests. Cluster-based permutation tests with 5000 Monte Carlo randomizations were applied to correct for multiple comparisons across time and sensors. Each iteration assigned random condition labels to each trial and extracted the cluster of sensors (*p* < 0.05, two-tailed) with maximal (negative or positive) summed statistics. To determine the precise time window of the N2 effect, a second analysis contrasted amplitudes from all time points of a pooled frontocentral channel (Fz, FC1, and FC2). To correct for multiple comparisons across time points, another permutation test with 5000 Monte Carlo randomizations was applied, based on the maximal (negative) statistic. To investigate the relationship between the N2 effect and forgetting, robust Pearson correlations were computed between the differential waveform of the pooled channel (mean Think minus No-Think amplitude within the significant window) and the mean SIF in both memory tests.

#### Time–frequency analyses.

Time–frequency representations (TFRs) were computed on data-padded wider epochs (−3000 to 5500 ms) to prevent edge filter effects. Epochs were convolved with 6 cycle and 3 cycle wavelets and then cropped to obtain 2–30 Hz spectral power between −500 ms prestimulus and 3000 ms poststimulus, in 50 ms × 1 Hz time–frequency bins. To further reduce the contribution of noise, participants' TFRs were normalized using a single-trial baseline correction method (Grandchamp and Delorme, 2011). First, power values of each time–frequency bin and channel were *z* transformed using the mean power and SD across all trials. After trial average, TFRs were converted into *z*-power change relative to baseline (−500 to 0 ms precue window) by subtracting the mean baseline *z*-power value from all time points of each frequency bin and channel. Within-condition relative power increases or decreases were determined by contrasting each time point against the mean baseline value at each frequency bin using nonparametric paired *t* tests. The *p*-values were computed through 5000 Monte Carlo randomizations. Then, false discovery rate procedure (Benjamini and Hochberg, 1995) was applied (*p* < 0.05) to correct for multiple comparisons across time–frequency bins and scalp locations. TFRs were contrasted between Think and No-Think conditions using cluster-based permutation tests with 5000 Monte Carlo randomizations to correct for multiple comparisons across time–frequency bins and scalp locations. Each iteration assigned random conditions labels to each trial and extracted the cluster of sensors and time–frequency bins (*p* < 0.05) with a maximal summed statistic. A similar statistical procedure was followed to compare TFRs between large and small N2 trials.

#### Source localization.

We created realistic three-shell boundary element models (BEMs) based on individual T1 MRIs. Each BEM consisted of three closed, nested compartments with conductivities of 0.33, 0.0042, and 0.33 S/m corresponding to skin, skull, and brain, respectively. To obtain the models, skull and brain binary images were obtained using the FieldTrip segmentation routine. Scalp voxels were first identified with the SPM8 “New Segment” algorithm (Ashburner and Friston, 2005) combined with an extended tissue probability map that includes eyeballs, the whole head, and the neck (for more details, see the procedure and code provided by Huang et al., 2013). The resulting scalp probability maps (including eyeballs) were smoothed and binarized. All binary images were manually corrected using MRI visualization software to better fit the anatomy and make them suitable for BEM computation (i.e., remove overlaps and irregularities). It was particularly critical to correct the scalp masks because EEG sensors were automatically classified as scalp tissue and would have produced bumpy models otherwise. Binary masks were used to create boundary meshes in FieldTrip using the iso2mech method with 10,000 vertices, which were smoothed afterward. Real EEG sensor coordinates were determined by hand from rendered models of raw scalp masks using MRI visualization software (ITK-SNAP). This was possible because EEG sensors were visible in the T1s and appeared as small bumps on the rendered models. TP9 and TP10 sensors (behind the ears) were often difficult to identify and were excluded from source localization analyses to reduce localization errors. A grid of source locations was defined in individual's brain space but corresponding to 1 cm grid MNI coordinates that were consistent across participants. To obtain individual coordinates, MRIs were normalized to the standard T1 template, using nonlinear transformations in SPM12. The inverse of this transformation was then applied to the template source grid obtained in FieldTrip. The leadfields were computed with OpenMEEG version 2.4 (Gramfort et al., 2010) called from FieldTrip, using international units (i.e., EEG amplitudes in volts; electrode and source-grid coordinates in meters).

To localize ERP and TFR effects, we used a linearly constrained minimum variance beamformer (Van Veen et al., 1997). The regularization parameter was set at 0.001% of the largest eigenvalue of the covariance matrices. To enhance the detection of N2 sources from less superficial brain areas such as those in dACC, we first subtracted the trial-averaged data (0.5–30 Hz) of the Think from No-Think condition. This method is called subtraction of epoch-averaged sensor data and is one of the subtraction techniques recommended to reduce interference from dominant sources common to two experimental conditions (Mills et al., 2012). The spatial filters were first computed within the time window showing significant amplitude differences at the sensor level (300–570 ms), and were then applied to the N2 and baseline time windows to obtain a power distribution map per participant. Participants' power distribution maps entered a group-level one-sample *t* test against zero. To correct for multiple comparisons, we applied the maximal statistic method using 5000 Monte Carlo randomizations implemented in FieldTrip (Oostenveld et al., 2011). We used a similar procedure to localize the sources within the time window of maximal amplitude differences in dACC ROI (548–708 ms). To determine whether source activity differences were task related, power distribution maps were contrasted with baseline power maps using paired-sample *t* tests and cluster-based statistics with 5000 randomizations. To determine the time window of maximal amplitude differences within dACC ROI, dipole momentum time courses were extracted for each Cartesian direction and averaged across trials for each condition. Think and No-Think amplitudes were compared with paired-sample *t* tests and cluster-based statistics, as described for sensor-level ERP analyses, to correct for multiple comparisons across time points, source locations, and orientations. To localize TFR effects, the spatial filters were computed over the whole epoch (−500 to 3000 ms) without averaging. For each predefined source location, dipole momentum time courses were extracted for each Cartesian direction and collapsed into a single trace after determining the principal component. TFRs were computed and *z* transformed as described for the sensor level. For statistical analyses, power values within the time–frequency window of interest were averaged and translated to brain maps. Contrasts were performed with paired-sample *t* tests and cluster-based statistics, as for ERP analyses. For ROI analysis, TFRs from all voxels within an ROI were identified and averaged across trials for each condition. Think and No-Think amplitudes were compared with paired-sample *t* tests and cluster-based statistics to correct for multiple comparisons across time–frequency bins and source locations. For control contrasts within hippocampal ROIs No-Think versus Think and Learned versus Not-Learned, we applied the sliding window approach. This method is a data-driven approach with two steps. In a first step, we performed cluster-based permutation tests for each time–frequency bin (50 ms × 1 Hz) within 0–2700 ms after cue onset and focusing on the theta frequency band (2–8 Hz). We included frequencies below the classic 4–8 Hz theta range for analyses involving hippocampal theta because this band is slower in humans than in rodents (Jacobs, 2014) and associative memory retrieval is dependent on 2–5 Hz hippocampal theta oscillations (Kota et al., 2020). Each test included a 300 ms sliding window of data, and identified significant clusters, correcting across time, frequency, and source locations (*p* < 0.05, one tailed). In the second step, another cluster-based permutation test with 5000 randomizations was performed including all consecutive time–frequency bins that were significant in the first step. This is done to test whether those consecutive time–frequency bins can be unified into a single cluster with consistent effects across time, frequency, and source locations (for more details, see Staudigl and Hanslmayr, 2013).

#### ROIs definition.

To test our a priori hypotheses, some analyses were restricted to the dACC, rDLPFC, and bilateral hippocampus. dACC and rDLPFC masks only included subregions that are commonly activated during action cancellation and memory inhibition, revealed by meta-analyses using fMRI data from stop-signal and TNT tasks (Guo et al., 2018; Apšvalka et al., 2022). Each mask comprised the cluster of voxels revealed by conjunction analyses combining the contrasts No-Think > Think and Stop > Go (Guo et al., 2018, their Fig. 32). Left and right hippocampal masks were constructed from a probabilistic map based on cytoarchitectonic delimitations derived from 10 postmortem human brains warped to the MNI template brain (Amunts et al., 2005). These maps contain the relative frequency with which a cerebral structure was present on each voxel of the anatomic MNI space. A binary mask was built from the region having a probability of ≥0.1 to be labeled as hippocampal [CA (Ammon's horn), DG (dentate gyrus), Subc (subiculum)] or entorhinal cortex (EC).

#### Granger causality analyses.

To investigate the effective connectivity and directionality of the information flow between our a priori selected ROIs, we performed GC analyses on EEG source activity. We computed spectral GC (Geweke, 1982) estimates within the 1 s time window right after the N2 (450–1450 ms) using the Fourier-based nonparametric approach (Dhamala et al., 2008) as implemented in FieldTrip (Oostenveld et al., 2011). We chose the nonparametric instead of the autoregressive approach because it does not require determination of the optimal model order for each participant. The 1 s window width was defined to be relatively short as to fulfill the stationarity assumption and have some temporal resolution, long enough to include sufficient data, and has been used in a previous application of the nonparametric approach in a similar context (Popov et al., 2018). To further approximate stationarity, we subtracted the event-related potential from the data before computing the Fourier transform for the whole spectrum (frequency smoothing, 2 Hz). Thereafter, the Fourier coefficients were used to compute the cross-spectral density matrix. This matrix was then factorized into the noise covariance matrix and the spectral transfer matrix, which are necessary for calculating GC (for more details, see Dhamala et al., 2008). To determine the statistical significance of the directionality of information flow between each pair of sources, we compared the magnitude of Granger coefficients (2–30 Hz) for both possible directions (from source 1 to source 2 and vice versa) and trial types (e.g., Think and No-Think) using cluster-based permutation ANOVAs and *t* tests. In addition, to confirm that the observed differences in GC values were caused by true directed relationships and not by differences in signal-to-noise ratio, we computed GC on the time-reversed source activities. After doing this control analysis, true causal interactions should show an inversion in the directionality of the information flow, whereas spurious interactions would appear as unchanged.

#### fMRI data preprocessing.

fMRI data were preprocessed using SPM12 software (Friston et al., 2007; Dhamala et al., 2008). Standard preprocessing steps were applied, including the following: (1) spatial realignment to correct for head movements; (2) slice timing; (3) coregistration of the structural to the functional images; (4) segmentation of the coregistered structural image (which performs spatial normalization to MNI space and generates a deformation field file); (5) normalization of the functional images applying the deformation field; and (6) spatial smoothing with a three-dimensional 6 mm full-width at half-maximum Gaussian kernel. As an additional control for head-movement artifacts, we excluded two data blocks in a participant because its mean head-movement regressors exceeded 4 mm in one of the orthogonal directions.

#### fMRI data analysis.

fMRI data acquired during the TNT phase was analyzed through general linear models (GLMs) in SPM12. Each data block for a given participant was modeled separately at the first level using a fixed-effects model. Then, each group analysis used a random-effects model. To identify the regions that were engaged or downregulated during retrieval suppression, we constructed a GLM containing three regressors of interest and one regressor of no interest. Regressors of interest were built with the onset times of the words presented in the three experimental conditions (Think, No-Think, and Perceptual Baseline). fMRI analyses were conditionalized, so Think and No-Think onset times corresponding to word pairs that participants failed to learn at study (Misses) were excluded from the main regressors and grouped in the regressor of no interest. Six further regressors contained the head-movement parameters obtained during spatial realignment and were included as covariates. First-level analyses calculated the main effects among the three conditions. For group-level analyses, contrasts between No-Think and Think, each relative to Perceptual baseline, were compared by means of paired-sample *t* tests. For whole-brain analyses, activations were considered significant if formed by clusters of at least 20 voxels with an uncorrected *p*-value <0.001. For dACC ROI analysis, a paired-sample *t* test was applied to averaged contrast values (*p* < 0.05).

#### ERP-informed fMRI analyses.

To determine whether and how (e.g., positive or negative modulation) BOLD responses of the ROI covaried with the EEG measures across trials, we chose a parametric design approach (Debener et al., 2006). For each EEG measure, we built a separate GLM. Each GLM extended the previously described GLM by including two additional regressors of interest and one of no interest. The regressors of interest were parametric modulator vectors for No-Think and Think conditions containing single-trial values of the corresponding EEG measure. The regressor of no interest gathered the onset times of all trials that were classified as artifacts during EEG preprocessing or that showed EEG measures larger or smaller than three SDs (i.e., these onset times were removed from the original main regressors). One of the parametric modulators was built from single-trial N2 amplitude values from the pooled frontocentral channel in the selected time window. To extract single-trial N2 amplitudes, an individual search window was first limited to participants P2 and P3 waveforms of the pooled channel ERP. Then, N2 latencies were defined as the minimum peak found within the individual search window for each trial. To help the peak detection algorithm, data were low pass filtered at 8 Hz. However, single-trial N2 amplitudes were obtained from the 0.5–30 Hz data, by averaging 100 ms windows centered on the N2 latencies. A second parametric modulator was built from mean theta (4–8 Hz) power values extracted from dACC ROI in the N2 time window. Other parametric modulators (i.e., theta power of hippocampal ROIs) were built from mean power values across all voxels within a cluster. We computed other GLMs for control purposes. In one case, we extracted the mean dipole momentum from dACC ROI within the N2 effect time window. To control for the sign of the source time courses and the correlation with BOLD, we run three separated GLMs, one for each Cartesian direction, and the resulting contrasts were averaged. The signs of the source time courses followed the same convention of the frontocentral channel in that window (i.e., more negative for larger N2 amplitudes, compared with small N2 amplitudes). For group-level analyses, contrasts of parameter estimates were averaged across all voxels within each ROI for each condition and subject. Within-condition and between-condition contrasts were assessed using one-sample, paired-sample, and independent-sample *t* tests, respectively. Whole-brain statistical maps were corrected for multiple comparisons using a cluster-based permutation approach. For each permutation, we used the same original GLM, but the order of the values in the parametric modulator (i.e., amplitude or latency) was randomized. As a result, the new parametric modulators contained the same values than the original regressor, but each value was assigned to an onset time of a different trial. This procedure was repeated 100 times in all participants (first-level fixed effects), which yielded 100 s-level fixed-effects analyses. We recorded the sizes of all clusters obtained from the resulting statistical maps after applying an initial threshold at *z* score >2.57 (uncorrected *p* < 0.005). Then, we generated a distribution with the cluster sizes, which enabled us to determine the largest cluster size leading to a significance level of *p* < 0.05. This cluster size was used as a threshold to correct the original statistical maps (Fouragnan et al., 2017).

## Results

The memory suppression task was a version of the TNT paradigm (Anderson and Green, 2001), as shown in Figure 2. First, participants (*n* = 24) encoded unrelated cue–associate word pairs and were trained to recall the associate given the cue. Then, participants entered the TNT phase, wherein they were presented with cues from studied items as reminders and directed to control the retrieval process, while we acquired simultaneous EEG-fMRI recordings. On each trial of the Think condition, participants received the cue word within a green frame and were asked to recall and think about its associate; on No-Think trials, by contrast, participants received the cue within a red frame and were asked to prevent the associate from entering consciousness. In a final phase, we performed two memory tests. On the SP test, participants received each cue word again and tried to recall its associate. On the IP test, participants instead received a novel category name and were asked to recall a word that belonged to that category from among the studied associates. We also tested memory for items that participants learned during the training phase, but that had not appeared during the TNT phase, providing a baseline estimate of retention for items that had neither been retrieved nor suppressed. Importantly, before entering the TNT phase, participants performed a memory test to identify the successfully learned cue–word associations. The mean (SD) proportions of learned word pairs for each condition were 74.48% (15.95) for Think, 79.95% (9.75) for No-Think, and 76.82% (13.48) for Baseline. The total number of trials corresponding to these learned associations across all sessions of the TNT phase were 141.96 (29.63) for Think and 152.08 (19.04) for No-Think.

### Behavior and confirmatory fMRI and EEG data analyses

**Figure 3.**
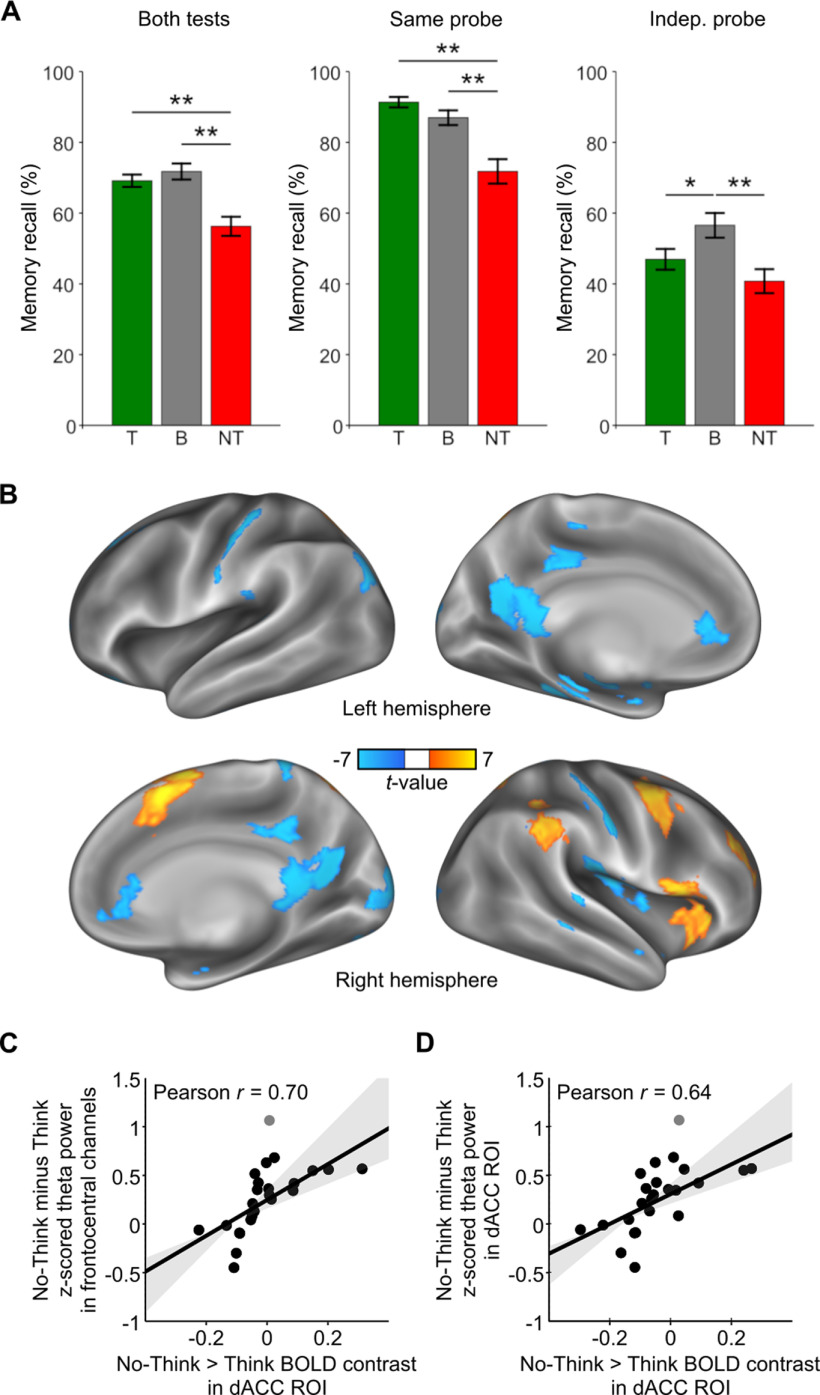
Behavioral and confirmatory fMRI and EEG results. ***A***, Percentage of items correctly recalled in the final memory tests using the same probes (middle panel) and independent probes (right panel). Mean values across both tests are shown in the left panel. Analyses were conditionalized on participants' recall before entering the TNT phase. Error bars show ± 1 SEM. Stars denote significant effects: **p < 0.001, *p < 0.05. T, Think; NT, No-Think; B, Baseline. ***B***, Whole-brain paired-sample contrast between No-Think and Think conditions. Hot colors indicate brain areas that are more active during retrieval suppression than during retrieval (No Think > Think), whereas cold colors indicate the opposite (No Think < Think). T-maps show values with *p* < 0.001 (uncorrected) and clusters with >20 voxels. ***C***, Scatter plot illustrates the positive robust correlation of No-Think versus Think effects in dACC BOLD signal and frontal-midline theta power increases in No-Think relative to Think, across participants. ***D***, Scatter plot illustrates the positive robust correlation between No-Think versus Think effects in dACC BOLD signal and dACC theta power increases in No-Think relative to Think, across participants.

We replicated key findings from previous studies (Anderson and Green, 2001; Anderson and Hulbert, 2021). As expected, participants recalled fewer associate words in the No-Think than in the Baseline condition (SP test: *t*_(23)_ = 4.65; *p* < 0.001; IP test: *t*_(23)_ = 4.74; *p* < 0.001; overall memory test: *t*_(23)_ = 6.16; *p* < 0.001; Fig. 3*A*). The below-baseline recall performance for No-Think items reflects SIF and confirms that participants successfully engaged inhibitory control mechanisms during retrieval suppression, which impaired memory. Although memory performance was lower on the IP test than on the SP test (test type effect: *F*_(1,23)_ = 94.52; *p* < 0.001), SIF generalized across both tests [Condition (No-Think vs Baseline) main effect: *F*_(1,23)_ = 24.00; *p* < 0.001; Condition (No-Think vs Baseline) * Test type interaction: *F* < 1; Anderson and Green, 2001; Anderson et al., 2004). In contrast, voluntary retrieval did not affect recall of Think items compared with baseline (overall memory test: *p* = 0.30; SP test: *p* = 0.15; but see Anderson and Green, 2001). Nevertheless, using different cues at recall than those studied and practiced was detrimental for retrieval (IP test: *t*_(23)_ = −2.55; *p* < 0.05), as has been previously reported, which is consistent with the encoding specificity principle (for a detailed discussion, see Paz-Alonso et al., 2009).

To confirm that suppressing associate words engaged dACC and rDLPFC, we analyzed fMRI data comparing the activation between NT and T trials from the TNT phase. Using a priori dACC and rDLPFC ROIs taken from a meta-analysis of 16 retrieval suppression studies (Apšvalka et al., 2022), we observed greater activity during retrieval suppression than during voluntary retrieval (dACC: +6, +23, +41; *p*(FWE) < 0.05, small volume corrected (SVC); rDLPFC: +36, +38, +32; *p*(FWE) < 0.01, SVC). With the opposite contrast (NT < T), we also confirmed decreased activity during retrieval suppression relative to voluntary retrieval in the hippocampus (left hippocampus: −33, −34, −10; *p*(FWE) < 0.001, SVC; right hippocampus: +24, −25, −13; *p*(FWE) < 0.05, SVC). Importantly, these deactivations were below the level observed in a perceptual baseline condition in which participants viewed unpaired single words presented within a gray frame (left hippocampus: −33, −31, −10; *p*(FWE) < 0.005, SVC; right hippocampus: +30, −25, −19; *p*(FWE) < 0.05, SVC), consistent with the view that retrieval suppression downregulates hippocampal activity (Depue et al., 2007; Gagnepain et al., 2017). In addition, an exploratory analysis using the overall contrast between No-Think and Think trials revealed BOLD activation patterns consistent with previous observations (for review, see Anderson and Hanslmayr, 2014; Anderson et al., 2016). Additional activations arose in mostly right-lateralized regions, including SMA, premotor cortex, inferior frontal gyrus, and parietal lobe, whereas additional deactivations occurred in brain areas that support the representation of visual memories, among other regions (Fig. 3*B*).

**Figure 4.**
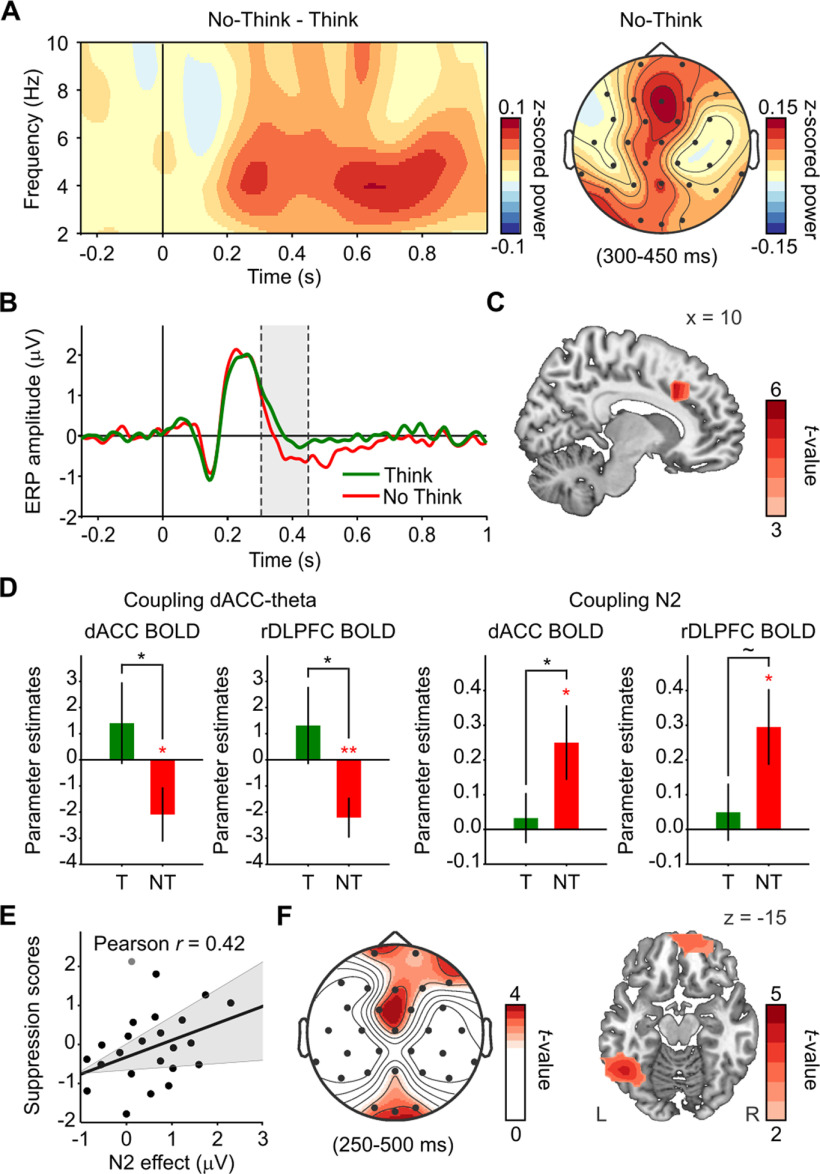
Early frontal-midline theta signals in dACC during retrieval suppression reflect proactive control. ***A***, Time–frequency (3 cycle wavelets) and scalp topographic maps showing increases in frontal-midline theta power during No-Think relative to Think. There was a cluster of frontocentral channels showing significant effects between 4–6 Hz and 250–800 ms (*p* < 0.05). ***B***, More negative amplitude of ERP waveform in No-Think (red) relative to Think (green; *p*-cluster < 0.05). Gray shadow indicates the time window 300–450 ms considered for N2 wave analyses. ***C***, Increased power in dACC sources during the suppression N2 (300–450 ms; *p* < 0.01, corrected with permutations and maximal statistic). ***D***, BOLD signals in dACC and rDLPFC were reduced in trials with increased dACC-theta power during retrieval suppression but not during retrieval. Similarly, BOLD signals in dACC and rDLPFC were reduced in trials with larger N2 signals during retrieval suppression. *p < 0.05; **p < 0.005; ∼*p* = 0.052. Error bars represent SEM. T, Think; NT, No-Think. ***E***, Individuals with larger the N2 showed more suppression-induced forgetting. Scatter plot shows a correlation between the N2 effect (ERP amplitude in Think minus No Think) and forgetting scores (*z*-SIF). Each participant's forgetting *z*-SIF was obtained by *z*-scoring its SIF index relative to the SIF of all participants receiving the same items in the same conditions (counterbalancing group). This transformation isolates suppression effects by correcting for irrelevant variability in forgetting because of differences in memorability of items across counterbalancing groups. ***F***, Scalp topographic and source maps showing increases in alpha power over left occipitotemporal regions (left fusiform gyrus) between 250 and 500 ms in trials with large N2 signals (*p*-cluster < 0.05). R, Right brain hemisphere; L, Left brain hemisphere.

We also verified that frontal-midline theta mechanisms were more engaged during memory suppression than during voluntary retrieval, reflecting higher overall control demands when cue-driven retrieval must be avoided. A nonparametric paired *t* test showed that theta power in the pooled frontocentral channel was enhanced in No-Think relative to Think, maximally between 250 and 800 ms (*t*-mean_(23)_ = 3.38, *t*-cluster = 104.6, *p*-cluster = 0.0036; but see Fig. 4*A*). This result is consistent with abundant evidence of frontal-midline theta power increases during tasks that involve cognitive conflict and the need to apply proactive or reactive control (Cavanagh et al., 2012; Cavanagh and Frank, 2014; Cavanagh and Shackman, 2015; Sauseng et al., 2019). Next, we directly tested the implicit hypothesis that suppression-related increases in frontal-midline theta power were related to more engagement of dACC. For this purpose, we extracted the mean No-Think versus Think BOLD contrast values for each participant from the voxels that were significant at the group-level dACC ROI analysis (*p*(FWE) < 0.05, SVC). Then, we correlated those values with No-Think minus Think *z*-scored theta power from the TFR of the pooled frontocentral channel (0–3 s, 4–8 Hz). This analysis was limited to the classic 4–8 Hz theta band, because control-related frontal-midline theta is typically expressed within this frequency range (Cavanagh et al., 2012; Cavanagh and Shackman, 2015). We found a significant positive correlation between dACC BOLD and frontal-midline theta No-Think versus Think effects, across participants (*p*-cluster = 0.0058). The overall robust Pearson correlation between these measures was significant when removing outliers (Fig. 3; ρ = 0.70, *t* = 4.57, *p* < 0.05). Finally, we additionally confirmed that the higher engagement of dACC during retrieval suppression was paralleled by increases in theta oscillatory power within this ROI. We performed another correlation analysis between mean No-Think versus Think BOLD contrast values and No-Think minus Think *z*-scored theta power from dACC TFR (0–3 s, 4–8 Hz). We found a significant positive correlation between BOLD and theta No-Think versus Think effects, across participants (*p*-cluster = 0.0026). The overall robust Pearson correlation between these measures was significant when removing outliers (Fig. 3*D*; ρ = 0.64, *t* = 3.90, *p* < 0.05).

### Early engagement of dACC theta control mechanism predicts reduced demands on dACC and rDLPFC for intrusion control

People who see a reminder to an unwanted thought and engage inhibitory control early enough may prevent the unwelcome memory from intruding. Achieving this form of proactive control requires a mechanism that detects the need for increased control on seeing a reminder. In natural settings, people may learn to identify warning features of stimuli that foreshadow unpleasant thoughts and use these features to initiate proactive suppression. We hypothesized that one of the key roles of dACC during motivated forgetting is to trigger this proactive mechanism to entirely prevent awareness of unwelcome content. This proactive mechanism may be initiated in the TNT task when participants process the red No-Think cues as task signals to stop retrieval. Related to this possibility, a previous EEG study gave participants advanced warning about whether each upcoming trial required suppression or retrieval; they found that anticipating the need for retrieval suppression increased theta power in dACC and left DLPFC sources in No-Think relative to Think trials within 500 ms after the suppression task warning (Waldhauser et al., 2015). Guided by previous findings (Cavanagh and Frank, 2014) and the overall positive correlation between dACC theta power and BOLD effects reported in the previous subsection, we used trial-by-trial modulations in theta power localized to dACC sources as an index of dACC engagement in upregulating inhibitory control during memory suppression and sought to relate this effect to BOLD signal in the dACC and rDLPFC. In contrast with the across-participants correlation analyses reported earlier, here we applied single-trial analyses to investigate how ongoing and temporally specific engagement in cognitive control (indexed by theta) relates to overall demands for memory control during suppression. To focus on proactive control, we measured dACC-theta within an early time window after cue onset and before the likely retrieval of the associates (∼500 ms; Staresina and Wimber, 2019). Although there is evidence of unconscious or prehippocampal associative memory retrieval earlier than 500 ms after the presentation of cues (Wimber et al., 2012; Koyano et al., 2016), many studies recording brain activity at different scales have shown that ∼500 ms marks the onset of hippocampal pattern completion preceding cortical reinstatement (500–1500 ms; for review, see Staresina and Wimber, 2019). Because intrusions are experienced in conscious awareness, intrusion-driven reactive control could only happen after this latency; so, in this sense we consider cognitive control before 500 ms as “early” or “proactive.” Our hypothesis implies two main predictions about how early dACC-theta power should relate to BOLD signals. First, if dACC is engaged in proactive control, we should expect elevated theta power during the early time window. However, provided that proactive control prepares the brain for mnemonic inhibition, facilitating retrieval stopping, successful engagement of this mechanism should have two effects. First, it should prevent intrusions, reducing aggregate demands on inhibitory control over the full 3 s duration of the trial (think of the adage: “a stitch in time, saves nine”); second, during intrusions, it should enable control to be more rapidly and robustly deployed to truncate recollection. Therefore, although theta activity is generated by the dACC, a robust early theta response should, paradoxically, predict less aggregate dACC BOLD signal during the trial, reflecting the diminished need for intrusion control. Similarly, a robust early dACC-theta response should lead to less rDLPFC BOLD signal over the duration of the trial.

To test whether these salutary effects of proactive control emerge, we measured trial-by-trial variations of EEG theta (4–8 Hz) power in dACC sources within an early time window where frontal-midline theta power started to increase in No-Think relative to Think (Fig. 4*A*). For consistency with the N2 analyses (see below), and to select activity prior the likely retrieval of the associates, we extracted mean activity within the 300–450 ms window. We then used this measure as a parametric modulator for an EEG-informed fMRI analysis. Consistent with our first prediction, trials with enhanced early dACC-theta power were associated with reduced BOLD signal in the dACC ROI, an effect specific to memory suppression (No Think, *p* = 0.03; No Think < Think, *p* < 0.05; Fig. 4*D*). To test the second prediction, we used the same parametric modulator but restricted the contrasts to the rDLPFC ROI. BOLD signal in the rDLPFC ROI was also reduced in trials with enhanced early dACC theta power, and this effect was also specific to suppression (No Think, *p* < 0.01; No Think < Think, *p* < 0.05; Fig. 4*D*).

We sought converging evidence for the role of proactive control in reducing demands on dACC and rDLPFC by focusing on a well established ERP component related to memory suppression. Previous studies have demonstrated the suppression N2 effect (more negative-going wave in No-Think than in Think at 350–450 ms), which appears to index the engagement of early inhibitory control during suppression; it correlates with the N2 effect of motor stopping (Mecklinger et al., 2009), with SIF, and with the consequent reduction in distressing intrusions of lab-analog traumatic memories (Streb et al., 2016). Other studies have found suppression N2 effects in a time window starting at 300 ms, which also shows item-specific forgetting effects (Bergström et al., 2009; Waldhauser et al., 2012). Taking into account its early latency and medial frontal topography, we hypothesized that this suppression N2 may be partly generated in dACC and reflect an aspect of the same frontal-midline theta mechanism that processes the need for control (Cavanagh and Frank, 2014) after seeing the No-Think cues. Replicating the aforementioned studies, mean ERP amplitudes during No-Think trials were significantly more negative than they were during Think trials between 300 and 450 ms (paired two-tailed *t* test: *t*-mean_(23)_ = −2.65, *p*-cluster = 0.0034). Differences were maximal between 327 and 450 ms at frontal, central, and right parietotemporal sensors. The N2 effect was localized at the right SMA (BA6: 10, 0, 60; *p* < 0.001), and supporting our hypothesis, at other frontal-midline regions including the dACC (peak voxel: 10, 20, 40; *p* < 0.001; Fig. 4*C*). To be consistent with how N2 is measured in other studies, we created a pooled frontocentral channel (Fz, FC1, and FC2) and compared again the amplitudes of No-Think and Think trials within the 300–450 ms window (Fig. 4*B*). This frontocentral channel showed peak differences between 332 and 348 ms (all *t*_(23)_ less than −2.70, *p*-corrected < 0.05), confirming that the N2 effect likely was generated before participants recollected the associate and agreeing with a proactive control role. If proactive control prepares inhibition in advance (Hanslmayr et al., 2009), it should allow participants to robustly and rapidly inhibit intrusions, yielding greater hippocampal downregulation and more forgetting. If so, people showing greater forgetting may show larger N2s. Consistent with this possibility, participants with a larger N2 effect (more negative going N2 in No-Think relative to Think trials) showed higher SIF scores (*r* = 0.42; *p* < 0.05; one outlier; Fig. 4*E*); indeed, only high forgetters showed differences in amplitude during this time window (332–348 ms, all *t*_(11)_ less than −2.88, *p*-corrected < 0.05) but not low forgetters (all *t*_(11)_ greater than −1.66, *p*-corrected > 0.24). Our results confirm previous findings and suggest that the N2 effect reflects early control processes in dACC that facilitate memory inhibition (Bergström et al., 2009; Mecklinger et al., 2009). We do not rule out, however, the existence of even earlier selective attentional processes that would enable individuals to disengage from further processing the reminders to unwanted memories, and limiting recollection without the need to engage inhibitory control (Bergström et al., 2007). We may not have found earlier ERP differences because we did not use the same methodological manipulation as was used in the previous study in which such early processes were found.

If the N2 reflects early inhibitory control, increases in this component, just as with dACC-theta power, should be negatively related to BOLD signal in both the dACC and rDLPFC. To test this, we extracted trial-by-trial variations of the N2 amplitude at the frontocentral channel. Then, we used this measure to build a parametric modulator for an EEG-informed fMRI analysis. Consistent with our hypothesis, trials with larger (more negative) frontocentral N2 amplitudes were accompanied by reduced BOLD signal in the dACC ROI (*p* < 0.05) specifically in No-Think trials (*p* < 0.05; Fig. 4*D*). Similarly, trials with larger (more negative) frontocentral N2 amplitudes during memory suppression were associated with reduced BOLD signal in the rDLPFC ROI (*p* < 0.05), although the differences relative to voluntary retrieval did not achieve significance (*p* = 0.052). Together, the timing of the N2 component and the negative relationship to the overall BOLD signal across the full duration of the trial suggest that the N2 effect, like dACC-theta, partially reflects proactive inhibitory control over memory. If this interpretation is correct, this proactive mechanism may contribute to stopping retrieval processes, reducing the occurrence of intrusions, and preempting any need for further engagement of dACC and rDLPFC during the suppression trial.

To further scrutinize this hypothesis, we tested whether the N2 was associated with EEG oscillatory markers of elevated cognitive control. First, we expected to link the N2 with early increases in frontal-midline theta activity; and second, we expected to find enhanced alpha/beta-band activity in regions involved or under top-down inhibitory control (Waldhauser et al., 2015; Castiglione et al., 2019; Fellner et al., 2020). To test this, we split TFRs of sensors on No-Think trials based on the amplitude of the frontocentral N2 as an index of proactive control. Trials with larger (more negative) N2 amplitudes showed, in addition to frontocentral theta power increases, increased alpha power relative to trials with smaller N2 amplitudes. Effects were maximal at frontal (4–8 Hz, 200–500 ms; 9–12 Hz, 350–450 ms; all *t* values > 2.46; *t*-cluster = 226.9; *p*-cluster = 0.01) and occipitoparietal sensors (9–12 Hz, 250–500 ms; all *t* values > 2.55, *t*-cluster = 132.8; *p*-cluster = 0.057; Fig. 4*F*). Alpha power differences were maximal in two clusters of brain sources, one including left temporal and occipital areas (peak voxel: −50, −50, 0, BA37; *t* = 4.54, *p*-cluster = 0.038) and another one maximal in right medial superior frontal gyrus (peak voxel: 10, 70, 10, BA10; *t* = 4.07, *p*-cluster = 0.019). These findings suggest a possible mechanistic link between frontal-midline theta and posterior alpha power increases (Waldhauser et al., 2015). One interpretation of this link is that preempting intrusion-related conflict via early proactive control might be partly achieved by increasing local inhibition in visual cortical areas and suppressing cortical memory representations (Gagnepain et al., 2014).

Together, the foregoing across-trial coupling findings suggest that the more that early control was engaged (as indexed by frontal-midline theta and N2), the less overall control demands arose throughout the trial, as reflected in reduced BOLD signal in rDLPFC and dACC. It is important to note that such relationships are not incompatible with the positive theta-BOLD correlations found in our across-participants analyses. We interpreted global increases in frontal-midline theta power as indicative of the higher need for cognitive control during memory suppression relative to voluntary retrieval, based on a well supported hypothesis about the role of this EEG marker (Cavanagh and Frank, 2014). However, recent findings indicate that, although on average frontal-midline theta power is higher for conditions that require more cognitive control (here No-Think) relative to control conditions (e.g., Think), and correlate with slower behavior, increased frontal-midline theta power also indexes the extent to which control is engaged (Eisma et al., 2021). Particularly, in trial-by-trial analyses focused within a condition, more theta predicts higher control engagement and faster responses on a task requiring inhibitory control (Cooper et al., 2019). Given these considerations, although we should expect more frontal-midline theta power in No-Think relative to Think, on average, we should also expect more frontal-midline theta power on trials where inhibitory control was more efficient in stopping retrieval. This means that, if early frontal-midline theta mechanisms were efficiently engaged, the relationship between transient theta power increases and the global demands for cognitive control by dACC and rDLPFC in the trial may appear inversed in across-trial EEG–fMRI coupling analyses.

### Delayed evoked responses in dACC and frontal-midline theta during suppression may reflect the detection of control demands caused by intrusions

**Figure 5.**
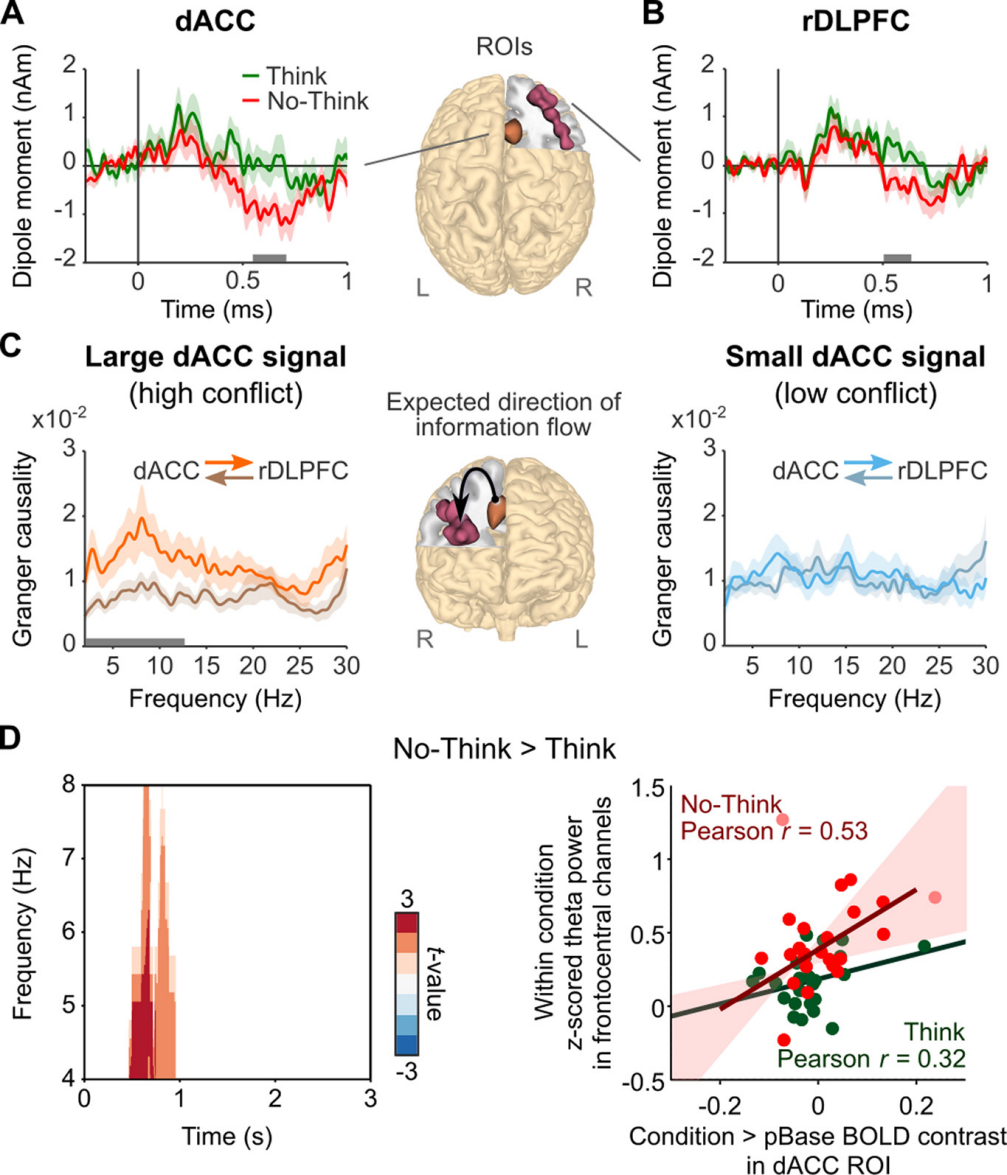
Time courses and Granger causality analysis of delayed dACC and rDLPFC effects. ***A***, ***B***, Mean evoked activity (0.5–30 Hz) in dACC (***A***) and rDLPFC (***B***) showing significant differences between each condition in a delayed time window (548–708 and 504–640 ms, respectively). Horizontal gray bars indicate the significant time windows (*p*-cluster < 0.05). EEG source time courses were reconstructed from the ROIs shown in the brain representations. ***C***, Granger causality spectra of information flow between dACC and rDLPFC sources (450–1450 ms) in trials associated with high and low conflict (large or small dACC activity in the 428–728 ms window, 0.5–8 Hz). In trials with more conflict-related activity, the information flow goes primarily from dACC to rDLPFC between 2 and 12.5 Hz. Horizontal gray bar indicates the significant frequency window (*p*-cluster < 0.05). Light shadowed areas represent SEM. ***D***, Time–frequency statistical map (left) illustrates that suppression-specific increases in frontal-midline theta, related to dACC engagement, occurred in a delayed time window. Scatter plot (right) illustrates the positive robust correlation between No-Think and Perceptual Baseline effects in dACC BOLD signal and frontal-midline theta power increases in No-Think, across participants. The equivalent analysis for Think was not significant. R, Right brain hemisphere; L, Left brain hemisphere.

When early control fails to suppress cue-driven retrieval, unwanted memories may intrude into awareness, creating cognitive conflict and driving higher demands on inhibitory control over memory (Levy and Anderson, 2012). We hypothesized that, in addition to its role in signaling early control demands in response to the task cues, dACC detects intrusions as “alarm” signals that indicate the need to further increase inhibitory control (Alexander and Brown, 2011). If intruding recollections trigger such a delayed control response, modulations in the evoked activity of dACC should arise after the latency where recollection could start (∼500 ms; Staresina and Wimber, 2019). To test this prediction, we extracted source time courses of the dipole momentum (0.5–30 Hz) from all voxels within our dACC ROI, for neural dipoles oriented in each Cartesian direction. Then, these source time courses were baseline corrected and averaged across trials of each condition (a similar procedure was followed to obtain sensor ERPs) to obtain the mean amplitude of evoked source activity for each voxel and orientation within the dACC ROI. Finally, we compared the mean amplitudes observed during No-Think and Think trials across all time points after cue onset (0–1 s). Accordingly, dACC amplitude was larger (and more negative going when projected to the sensor space) during No-Think trials than during Think trials between 548 and 708 ms (*t*-mean_(23)_ = −2.51, *t*-cluster = −793.6, *p*-cluster = 0.0064; Fig. 5*A*). Amplitude differences were only significant in dACC sources oriented radially to the top of the head, consistent with the topography of ERPs related to novelty, conflict, or error processing (Cavanagh et al., 2012). These ERPs are thought to be part of the same frontal-midline theta mechanism for realizing the need for cognitive control (Cavanagh and Frank, 2014); therefore, we expected that these delayed control signals in dACC particularly would involve theta-band activity. To test this, we first recomputed the mean source amplitudes as described above, but previously filtering the EEG signals to include either delta/theta band (0.5–8 Hz) or alpha/beta band (8–30 Hz). Indeed, mean amplitude differences were explained by delta/theta band (428–728 ms, *t*-mean_(23)_ = −2.51, *t*-cluster = −1511, *p*-cluster = 0.0046) and were not significant for alpha/beta band (all *p*-clusters > 0.33). Then, we investigated whether suppression-specific engagement of dACC would particularly enhance delayed frontal-midline theta oscillatory power. We performed within-condition correlations by extracting Think versus Perceptual baseline and No-Think versus Perceptual baseline mean BOLD contrast values from the dACC ROI (*p*(FWE) < 0.05, SVC). We correlated those values with corresponding frontocentral TFR *z*-scored theta power and compared the correlations between conditions (using FieldTrip function ft_statfun_depsamplesregrT) at all time points of the 3 s trial. We found that only between 464 and 956 ms, the correlation between BOLD and theta power increases in No-Think was significantly higher than the correlation in Think (*p*-cluster = 0.0286; Fig. 5*D*, left); no effect was found later in the trial. Within the significant window, we found a robust Pearson correlation between these measures in No-Think (ρ = 0.53, *t* = 2.93, *p* < 0.05) but not in Think (ρ = 0.32, *t* = 1.58, *p* > 0.05; Fig. 5*D*, right). The time window of this effect is consistent with our hypothesis that frontal-midline theta mechanisms may have been additionally recruited at the time the first intrusions could have been experienced in awareness (i.e., after the beginning of hippocampal pattern completion and following cortical reinstatement).

If intruding recollections drive these delayed dACC-evoked responses, these signals should be more evident for individuals who experience more intrusions. In healthy samples, participants who exhibit little suppression-induced forgetting show more intrusions during No-Think trials and diminished engagement of top-down inhibitory control over the hippocampus (Levy and Anderson, 2012; Gagnepain et al., 2017; Mary et al., 2020), paralleling deficient suppression-induced forgetting and enhanced intrusive symptoms in post-traumatic stress disorder (Catarino et al., 2015; Mary et al., 2020). If low suppression-induced forgetting participants experience more intrusions, delayed dACC-evoked responses may be greater in low forgetters. Agreeing with this, only low forgetters showed more negative amplitudes in the No-Think condition than the Think condition between 552 and 704 ms (*t*-mean_(11)_ = −2.68, *t*-cluster = −769.8, *p*-cluster = 0.0044). In contrast, high forgetters showed no reliable amplitude differences in this later window (no amplitude differences crossed the cluster formation alpha threshold), possibly reflecting both the reduced frequency of intrusions and their rapid truncation by a robust inhibitory control response (Levy and Anderson, 2012). However, these effects were not significantly different between groups (interaction: *p*-cluster = 0.053). These findings are in line with our hypothesis that dACC is also involved in the late adjustment of inhibitory control when unwanted memories emerge. Moreover, they are consistent with the view that late evoked theta responses in dACC reflect cognitive conflict and control demand signals driven by intrusions.

### Delayed dACC responses during suppression trigger increased communication with rDLPFC through a theta mechanism

Once an intrusion is detected, the need for increased control should be transmitted to prefrontal areas that implement mnemonic inhibition. We hypothesized that dACC communicates an intrusion-control signal to rDLPFC through a mechanism of interareal neural coupling mediated by theta-band activity (Cavanagh and Frank, 2014; Smith et al., 2019). To test this idea, we first investigated whether rDLPFC-evoked activity showed modulations reflecting the reception of dACC signals. We reconstructed source time courses (0.5–30 Hz) from all voxels within the rDLPFC ROI and compared their mean amplitudes across No-Think and Think trials for all time points after cue onset (0–1 s). rDLPFC amplitude was more negative in No-Think than in Think trials at latencies overlapping the dACC effect associated with putative intrusion control (sources with left–right orientation: 408–588 ms, *t*-mean_(23)_ = −2.56, *t*-cluster = 1.374, *p*-cluster = 0.008; sources with inferior–superior orientation: 504–640 ms, *t*-mean_(23)_ = −2.53, *t*-cluster = −1.425, *p*-cluster = 0.016; Fig. 5*B*). Then, we performed a whole-brain source analysis within the delayed control window of dACC (0.5–8 Hz; 428–728 ms) to verify that these modulations were regionally specific and not caused by stronger nearby sources. We expected that, if dACC and rDLPFC were particularly engaged in control processes triggered by intrusions, these regions should show activity differences significantly larger than those shown before cue onset. Agreeing with this, two frontal clusters exhibited above-baseline effects: one in the right hemisphere comprising superior frontal gyrus, middle frontal gyrus, and ACC (peak voxel: 30, 30, 40, BA8; *t* = 3.75, *p*-cluster = 0.0086); and another in the left hemisphere comprising inferior frontal gyrus and insula (peak voxel: −50, 20, 0, BA47; *t* = 3.84, *p*-cluster = 0.037). We confirmed that dACC and rDLPFC ROIs showed significant effects (*p* < 0.05, corrected with permutations and maximal statistics).

Finally, we looked for functional coupling between dACC and rDLPFC, indicating the transmission of the control demands within the delayed control window. We investigated the directionality of the interaction between dACC and rDLPFC by applying nonparametric GC analyses to a 1 s window right after the N2 450-1450 ms, to exclude effects because of this ERP component. In trials with larger negative evoked activity in dACC, the information flow in the direction of dACC to rDLPFC was higher than that from rDLPFC to dACC for theta and alpha frequency bands (2–12.5 Hz, *p* = 0.0064, corrected with cluster-based permutation test). In trials with smaller dACC signals, there was no preferred direction in the information flow (*p*-cluster > 0.44; interaction effect, *p*-cluster = 0.09; Fig. 5*C*). Our results support our hypothesis, showing that dACC and rDLPFC were maximally engaged around the time that unwanted memories may have been retrieved. Strikingly, stronger responses in dACC generated Granger causal influences on rDLPFC activity within the theta and alpha frequency bands, consistent with a process communicating the need to intensify memory control.

### Early control processes involving dACC contribute to later hippocampal downregulation

**Figure 6.**
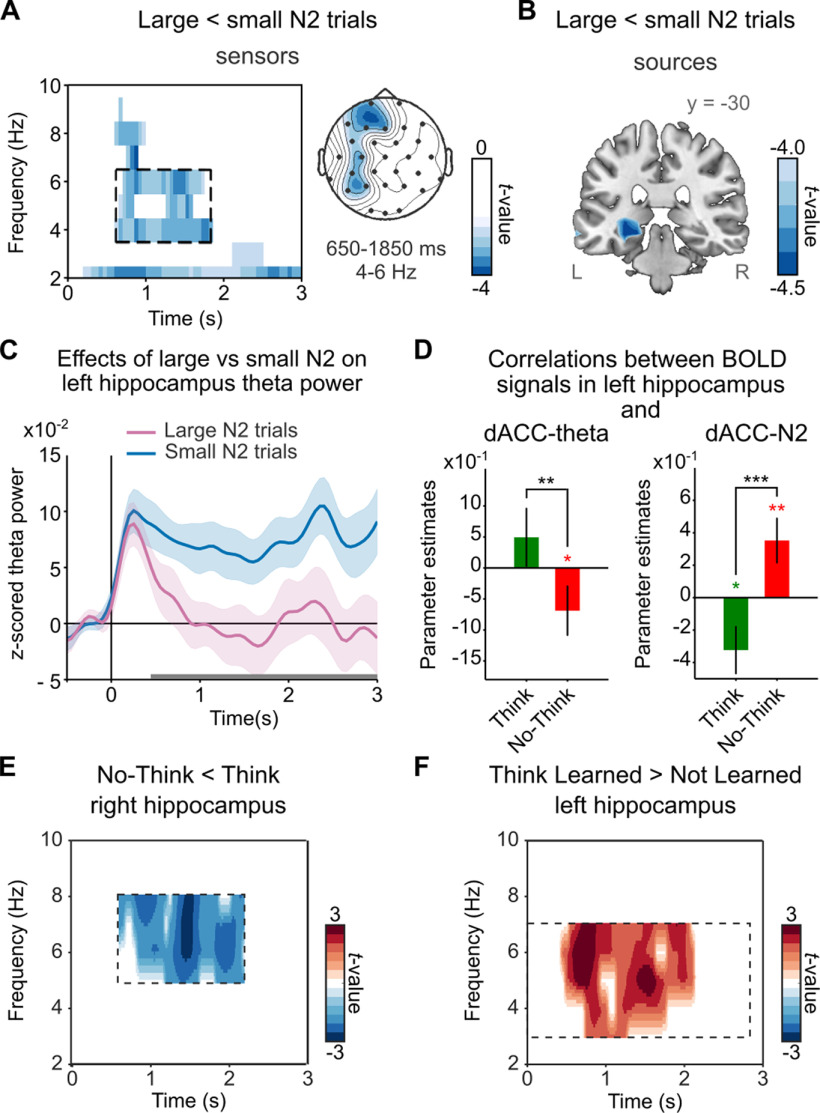
Effect of early control on later theta power activity and BOLD signals in the hippocampus. ***A***, TFRs from all sensors showing decreased theta power in trials associated with large N2 signal (putative strong proactive control) relative to trials with small N2 signal (putative weak proactive control; *p*-cluster < 0.05). The right panel shows the scalp topography of the effects in the framed time window (4–6 Hz, 650–1850 ms). ***B***, Sources of the peak theta power decreases were localized in left hippocampus (*p*-cluster < 0.05). ***C***, ROI average of hippocampus *z*-scored power time courses for trials associated with large or small N2 signals (putative strong or weak proactive control). Horizontal gray bars indicate the time window of significant differences between conditions in the ROI time–frequency analysis (*p*-cluster < 0.05). ***D***, BOLD signals in the hippocampus were reduced in trials with increased dACC theta power (300–450 ms) during retrieval suppression but not during retrieval. Similarly, BOLD signals in the hippocampus were reduced in trials with larger (more negative) dACC N2 amplitudes during retrieval suppression but not during retrieval. *p < 0.05; **p < 0.005; ***p < 0.0005. Light shadowed areas (***C***) and error bars (***D***) represent the SEM. ***E***, TFRs from all sources within right hippocampal ROI showing decreased theta power in No-Think relative to Think (*p*-cluster = 0.0258). ***F***, TFRs from all sources within left hippocampal ROI showing increased theta power in Think Learned items relative to Think Not-Learned items (*p*-cluster = 0.0238). Boxes with dashed lines (***E***, ***F***) indicate the time–frequency window included in step 2 of the sliding window statistical approach. R, Right brain hemisphere; L, Left brain hemisphere.

An intruding memory may not endure very long in awareness if inhibitory mechanisms already have been prepared when intrusions occur. We hypothesized that proactive control early in the trial would facilitate the later downregulation of memory-related networks, and that theta oscillatory activity associated with retrieval should reflect the impact of suppression (Waldhauser et al., 2015). We focused on theta-band activity because abundant animal and human literature shows that retrieval depends on hippocampal–cortical synchronization supported by the theta rhythm (Fuentemilla, 2018; Staresina and Wimber, 2019; Herweg et al., 2020). Noninvasive studies in humans indicate that retrieval is characterized by increased oscillatory power (Düzel et al., 2003; Osipova et al., 2006) and long-range phase synchronization in the theta band (Guderian and Düzel, 2005; Fuentemilla et al., 2014). Moreover, intracranial EEG recordings in humans (Kota et al., 2020) reveal that 2–5 Hz theta oscillatory power increases and phase reset in the hippocampus are selectively associated with successful memory retrieval. Based on these considerations, we expected that stopping retrieval by early control would reduce theta-band power in the hippocampus. We tested this hypothesis by median-splitting No-Think trials by their N2 amplitude (our hypothesized index of proactive control) and then looking for reflections of suppressed activity in sensor TFRs by applying the contrast larger < smaller N2 amplitude. Indeed, trials with larger N2 amplitudes showed reduced theta oscillatory power between 650 and 1850 ms after cue onset in left frontal, central, and parietal sensors, relative to those with smaller N2 amplitudes (Fig. 6*A*; 4–9 Hz; all *t* values less than −2.42; *t*-cluster = −493.9; *p*-cluster < 0.001). In trials with smaller N2 amplitudes (where proactive control may have been reduced) theta oscillatory activity persisted above baseline levels for the whole epoch. Consistent with our hypothesis, the greatest reduction in theta was localized in the left hippocampus (peak voxel: −30, −30, −10; *t* = −4.56; *p*-cluster = 0.0038; Fig. 6*B*), but both hippocampi showed that theta power decreases in ROI TFR analyses (left hippocampus: all *t* values less than −1.97; *p*-cluster < 0.005; right hippocampus: all *t* values less than −1.84, *p*-cluster = 0.014; Fig. 6*C*).

**Figure 7.**
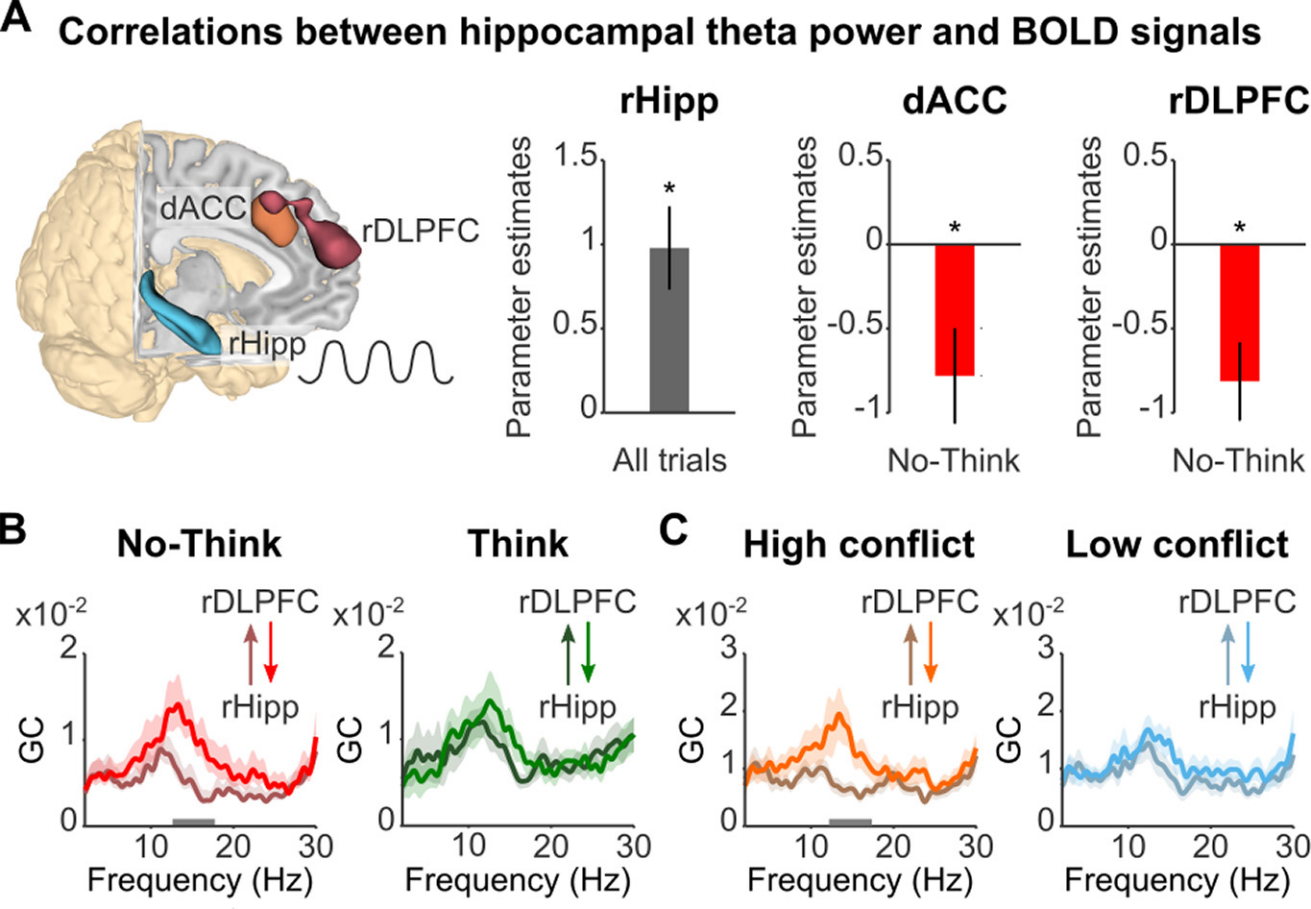
Downregulation of hippocampus by late control. ***A***, EEG-informed fMRI parametric analyses using hippocampal theta power as regressor. Voxels in dACC (orange) and rDLPFC (burgundy) showing significant (*p* < 0.005) negative correlation with theta power reconstructed from sources of right hippocampus (rHipp) ROI (cyan). Bar plots show that theta power in the right hippocampus was (1) positively coupled to BOLD signal in this region and (2) reduced in trials with increased dACC and rDLPFC BOLD signals during retrieval suppression. ***B***, ***C***, Granger causality spectra of information flow between rDLPFC and right hippocampus sources in No-Think and Think (***B***) and trials associated with high and low conflict (large or small dACC activity in the 548–708 ms time window; ***C***). In No-Think and in trials with more conflict-related activity, the information flow goes primarily from rDLPFC to right hippocampus in low beta band. Horizontal gray bars indicate the significant frequency windows (*p*-cluster < 0.05). Error bars (***A***) and light shadowed areas (***B***, ***C***) represent SEM. GC, Granger causality.

These findings indicate that early control processes contributed to the later stopping of hippocampal retrieval, consistent with a potential benefit of proactive control. To verify the localization of these effects to the hippocampus, we performed several additional analyses. First, we related EEG theta activity reconstructed from hippocampal sources to hippocampal BOLD signal. EEG-informed fMRI analysis using mean hippocampal theta power extracted from the larger < smaller N2 effect (4–6 Hz, 650–1850 ms) as a parametric modulator revealed a positive correlation between theta power and hippocampal ROIs BOLD signals (right: *p* < 0.005, uncorrected; SVC, *p*(FWE) = 0.067; left: *p* < 0.05, uncorrected; Fig. 7*A*) across all trials, consistent with the hypothesis that our hippocampal theta sources reflected hippocampal processing. Next, we sought converging evidence of proactive mnemonic control by relating trial-by-trial variations in early dACC-theta power to hippocampal BOLD signals during retrieval suppression. We found that trials with increased dACC-theta power showed stronger hippocampal deactivations, specifically during memory suppression (Fig. 6*D*). We confirmed that, with an alternative EEG-informed fMRI analysis, more negative N2 amplitudes, extracted trial-by-trial from the dACC ROI, also correlated with reduced hippocampal BOLD signals, again specifically during memory suppression (SVC, *p*(FWE) < 0.05; Fig. 6*D*). Together, these parallel findings strongly support our hypothesis that dACC triggers proactive inhibitory control to stop the retrieval of unwanted memories by the hippocampus.

The delayed latency (650–1850 ms) of the theta suppression effect found in trials with larger (relative to smaller) N2 amplitudes suggests that although early control processes may help to prevent intrusions, they also enhance readiness to purge intruding thoughts from awareness when they do occur, overlapping with and likely facilitating hippocampal downregulation by reactive control processes. To examine whether a reactive hippocampal downregulation arose, we analyzed the time course of hippocampal theta activity as an index of hippocampal retrieval. Specifically, we examined those trials associated with higher putative intrusion control, to test for the presence of (1) an early increase in hippocampal retrieval activity that might reflect the initial involuntary retrieval, followed by (2) hippocampal activity suppression. To test this, we divided No-Think trials according to the power of the intrusion-control signals they exhibited in dACC (2–8 Hz, 428–728 ms). In trials with larger intrusion control signals, hippocampal theta power (2–8 Hz) was increased only during the first half of the epoch (left: 300–1600 ms, *t*-mean = 3.16, *t*-cluster = *p*-cluster < 0.001; right: 0–1700 ms, *t*-mean = 2.81, *t*-cluster = *p*-cluster < 0.01), relative to trials showing smaller intrusion control signals. The fact that no significant differences emerged in hippocampal theta power during the second half of the epoch is consistent with reactive suppression of hippocampal theta in response to an intrusive recollection.

To further validate the hippocampal theta source reconstruction achieved in this study, we sought to confirm that we observed expected benchmark effects from two additional contrasts: the No-Think versus Think and the Learned versus Not-Learned contrasts. If our source reconstruction is valid and theta truly reflects hippocampal retrieval, we should find (1) less hippocampal theta overall during No-Think than Think trials, because they should differ in the amount of retrieval, and (2) more hippocampal theta for learned Think items (which can be retrieved) than for not learned Think items (which cannot be retrieved). Accordingly, we found theta (*z*-scored) power decreases in No-Think relative to Think in both hippocampal ROIs, although only the right hippocampus remained significant after correction for multiple comparisons (*p*-cluster = 0.0258, 600–2200 ms, 5–8 Hz; Fig. 6*E*). Both the latency (relative to the reminder) and frequency range of this effect match those of equivalent theta suppression observed at sensor level (Waldhauser et al., 2015). For the Learned versus Not-Learned analysis, the Learned condition comprised all trials belonging to Think items that were successfully learned at the memory test performed before the TNT phase (pretest) and successfully remembered at both final memory tests. The Not-Learned condition comprised all trials belonging to Think items that were not learned at the pretest and not remembered in at least one of the final memory tests. Because some participants had none or too few trials in the Not-Learned condition, we restricted the sample to those participants with a minimum of 12 trials in the Not-Learned condition (one trial per TNT repetition). The Learned versus Not-Learned contrast (*n* = 20) showed a significant theta (*z*-scored) power increase in Learned relative to Not-Learned items in both hippocampal ROIs (right: *p*-cluster = 0.0168, 0–2200 ms, 3–8 Hz; left: *p*-cluster = 0.0238, 450–2100 ms, 3–7 Hz; Fig. 6*F*). Together, these findings corroborate our assumption that our hippocampal theta source reconstructions were influenced by hippocampally mediated retrieval activity; in doing so, they reinforce the conclusion that engaging early control facilitates the later suppression of theta activity within the hippocampus that drives the retrieval of intruding memories.

### RDLPFC downregulates the hippocampus in response to late dACC control signals

The foregoing findings suggest an account in which early and delayed control mechanisms during memory suppression contribute to hippocampal downregulation, which is expressed as theta power decreases. By this interpretation, when early control fails to prevent unwanted memories from intruding, dACC generates signals indicating the need for intrusion control. These signals may dynamically adjust inhibitory control mechanisms in the rDLPFC to downregulate hippocampal activity (Benoit et al., 2015) to purge the intruding memories. Consistent with this intrusion-purging hypothesis (Levy and Anderson, 2012), during suppression, increased BOLD signals in rDLPFC and dACC were coupled to decreased theta power in bilateral hippocampal sources after the likely onset of recollection (rDLPFC and right hippocampus: SVC, *p*(FWE) = 0.02; left hippocampus: *p* < 0.005, uncorrected; dACC and right hippocampus: SVC, *p*(FWE) = 0.01; theta power was extracted from the time–frequency window that showed significant decreases in trials with larger, relative to smaller, N2 amplitudes: 650–1850 ms, 4–6 Hz), and the latter theta suppression effect was itself associated with hippocampal downregulation (Fig. 7*A*). These findings suggest that hippocampal theta power decreases (observed after retrieval onset) may arise from increased prefrontal inhibitory control.

To examine whether rDLPFC and dACC activity during putative intrusion control may be causally related to hippocampal theta decreases, we used nonparametric GC focusing on a 1 s window after the N2 (450–1450 ms). First, we investigated the direction of information flow between rDLPFC and hippocampus. This analysis revealed a higher top-down than bottom-up information flow during retrieval suppression in the theta band (rDLPFC to left hippocampus: 2.0–6.5 Hz, *p* = 0.021, corrected with cluster-based permutations; interaction effect: 2-5.6 Hz, *p*-cluster < 0.05) and in the low beta band (rDLPFC to right hippocampus: 12.6–17.8 Hz, *p* = 0.023, corrected with cluster-based permutations; Fig. 7*B*). Importantly, the increased top-down information flow in the beta range specifically arose during trials with larger control signals in dACC (12.2–17.4 Hz, *p*-cluster = 0.023; interaction effect: 12.3–15.4 Hz, *p*-cluster = 0.023; Fig. 7*C*), consistent with the possibility that dACC triggered the rDLPFC top-down mechanism that caused hippocampal downregulation. To test this idea, we identified trials in which the suppressive impact of the hippocampus was clear and examined the influence of dACC on rDLPFC; we split No-Think trials according to the mean theta power in hippocampal sources within the time–frequency window that showed significant decreases in trials with larger (relative to smaller) N2 amplitudes (650–1850 ms, 4–6 Hz) and computed GC analyses within that window. We found that trials with reduced hippocampal theta showed a higher information flow in the theta band (4–9.73 Hz, *p*-cluster < 0.05) from dACC to rDLPFC than in the opposite direction, consistent with a role of dACC in triggering elevated control. Together, these findings point to a late influence of the rDLPFC on the hippocampus and suggest that beta oscillations mediate a reactive top-down inhibitory control mechanism triggered in response to intrusions detected by the dACC.

Top-down inhibitory control from the rDLPFC to the hippocampus may facilitate forgetting of the suppressed memories (Gagnepain et al., 2014; Benoit et al., 2015; Anderson and Hulbert, 2021; Apšvalka et al., 2022). We tested this possibility by dividing participants into those who showed higher or lower SIF scores. High forgetters showed a greater top-down than bottom-up information flow from rDLPFC to right hippocampus for suppression items that they successfully forgot, relative to those that they remembered (19.8–23.0 Hz, *p*-cluster = 0.031). In contrast, less successful forgetters showed the opposite pattern, with a higher top-down than bottom-up information flow for suppression items that they later remembered (11.6–17.6 Hz, *p*-cluster = 0.006). These findings suggest that in low forgetters, the reactive engagement of top-down inhibitory control in response to intrusions is related to persisting memory for intruding thoughts.

## Discussion

Our findings reveal a central role of dACC in triggering inhibitory control that causes motivated forgetting. The data suggest that dACC signals the need for inhibition proactively, in response to environmental cues, or reactively, to counter intrusive thoughts. Two key findings support the proactive role of dACC in preventing retrieval. First, frontal-midline theta mechanisms partly originated in dACC and emerged at early latencies before the likely onset of episodic recollection; and second, these mechanisms were associated with reduced BOLD signals and theta power in the hippocampus, consistent with reduced retrieval activity. Our findings suggest that rapidly detecting the need for suppression from an environmental signal (e.g., the task cue) engages proactive control by dACC to prevent intrusions. Consistent with this interpretation, trials with increased early theta signals from dACC were accompanied by lower BOLD activations of dACC and rDLPFC, reflecting lower demands on prefrontal control when recollection was quickly mitigated. Thus, rapid early control was beneficial; as the adage says, “a stitch in time, saves nine.” These effects echo those of a prior fMRI study investigating the benefits of forgetting on neural processing during a retrieval-induced forgetting task (Kuhl et al., 2007). That study showed that, as competing memories were suppressed across retrieval practice trials, the demands to detect and overcome conflict were reduced, and so activations in ACC and lateral PFC declined. Similar conflict reduction benefits (associations between successful memory control and reduced conflict processing) have been shown in a range of studies (Anderson and Hulbert, 2021).

Importantly, our results suggest that proactive control did not simply prevent retrieval, but also facilitated forgetting. Several observations support this conclusion. First, individuals with larger suppression N2 showed superior suppression-induced forgetting, complementing earlier findings linking the suppression N2 to better SIF (Bergström et al., 2009), and to fewer distressing intrusions after analog trauma (Streb et al., 2016). Second, the N2 was partly generated in dACC and associated with early (<500 ms) frontal-midline theta power increases, a common activity pattern reflecting the detection and communication of the need for control to DLPFC (Cavanagh and Frank, 2014). These mechanisms could trigger early inhibitory control targeting regions supporting memory. Indeed, N2-associated early increases in alpha oscillatory power (a typical correlate of cortical inhibition; Klimesch, 2012) in left fusiform gyrus may be an example: given that downregulation of this region by DLPFC during suppression is known to disrupt visual memory traces of the associates (Gagnepain et al., 2014), elevated local alpha power may both limit the reinstatement of associated words and suppress their memory traces, resembling the downregulation of item-specific memories (Fellner et al., 2020). We also linked early control signals to decreased hippocampal theta power in trials with larger (relative to smaller) N2 amplitudes during a later time window (650–1850 ms). Theta power decreases in the medial temporal lobe have been previously linked to successful suppression-induced forgetting (Waldhauser et al., 2015). The current hippocampal theta modulations started at ∼300 ms, suggesting that early control affected later hippocampal-cortical theta networks. Given that hippocampal pattern completion appears to begin at ∼500 ms after cue onset (Staresina and Wimber, 2019), the 650–1850 ms window offers an opportunity for rDLPFC action over the hippocampus (Depue et al., 2007; Benoit and Anderson, 2012; Benoit et al., 2015; Gagnepain et al., 2017; Apšvalka et al., 2022) to inhibit memory traces, stopping intrusions. Indeed, we found a significant causal influence of rDLPFC on both hippocampi within 450–1450 ms only during suppression. Together, these findings suggest that proactive control facilitates forgetting by increasing inhibition in regions where memories would be reactivated, and, by magnifying the impact of intrusion-control mechanisms.

Although our evidence for early control is compatible with the proactive prevention of retrieval, we cannot rule out the possibility that this early control process may be triggered reactively, perhaps in response to a rapid, nonrecollective form of retrieval. The red cues used in our study did not merely signal the need for retrieval stopping but were also reminders of the associated words. These cue words may have elicited an early nonconscious or prehippocampal reactivation of the associated memory. This reactivation may have been detected by dACC. If so, the occurrence of the N2 or frontal-midline theta before 500 ms could be reactive control as well (Hellerstedt et al., 2016). We note, however, that even the process we had proposed at the outset required the detection of “warning signals” in the environment to set in motion proactive mechanisms, and the recognition of cues as warning signals would itself be a form of rapid retrieval. Thus, the fact that control is initiated by the retrieval of something—either the warning status of a percept or preconscious traces of the target memory—is not incompatible with it serving a proactive control role. In this instance, control would be accurately described as proactive with respect to the prevention of intrusive recollections.

Critically, however, dACC also triggered inhibitory control in a later time window during which unwanted memories could have been involuntarily retrieved. Our findings suggest that dACC detects conflict caused by intruding memories and communicates the need for control to rDLPFC, which in turn increases top-down inhibition over the hippocampus. Supporting this conclusion, we found, during retrieval suppression, peak dACC and rDLPFC source activities in a delayed time window, coinciding with the likely onset of conscious retrieval. dACC activity between 552 and 704 ms was particularly large for low forgetters, suggesting that these signals reflected intrusion-related conflict. Moreover, dACC effects were in delta/theta frequencies, which is a preferred rhythm for the coherent firing of dACC and DLPFC neurons during conflict processing, and for cross-areal coordination to implement control (Smith et al., 2019). Precisely, using this dACC delta/theta amplitude as a proxy for conflict, we found that high conflict was associated with high information flow from dACC to rDLPFC in delta/theta band and from rDLPFC to right hippocampus in beta band. The greater the activation in dACC and rDLPFC, and the stronger their theta-mediated communication, the stronger was the implementation of control over hippocampal activity, reflected by decreases in hippocampal theta.

Prefrontal control over hippocampal activity, once triggered by dACC, appears to be achieved via beta-phase synchronization. During retrieval suppression, rDLPFC increased its communication with the right hippocampus in the low beta band, an effect not found during retrieval trials. This higher information flow, moreover, arose specifically on high-conflict trials, consistent with the need to intensify top-down control to purge intrusions from awareness. Elevated beta-mediated communication with the hippocampus during intrusions integrates prior evidence that had separately illustrated the importance of intrusions and beta activity in inhibitory control over memory. On the one hand, fMRI studies have found that intrusions elicit greater rDLPFC activation (Benoit et al., 2015), stronger hippocampal suppression (Levy and Anderson, 2012; Benoit et al., 2015; Gagnepain et al., 2017), and more robust frontohippocampal interactions to suppress retrieval (Benoit et al., 2015; Gagnepain et al., 2017), although the oscillatory mechanisms of these intrusion effects were not established. On the other hand, the importance of beta oscillations to memory inhibition has grown increasingly clear, but this activity has not been linked to reactive control over intrusions. For example: (1) at the scalp level, retrieval suppression increases long-range synchronization in low beta frequency band (15–19 Hz; Waldhauser et al., 2015), suggesting that this rhythm (together with alpha) might mediate top-down control; (2) at the intracranial level, directed forgetting instructions elicit DLPFC–hippocampal interactions in the low beta range (15–18 Hz; Oehrn et al., 2018), with greater information flow from DLPFC to hippocampus when people were instructed to forget an item, but not when instructed to remember it; and (3) stopping actions and stopping retrieval elicit a common right frontal low beta component (Castiglione et al., 2019). Together with the current results, these findings point to a key role of beta oscillations in the top-down control over hippocampal processing by rDLPFC, a demand that is especially acute during the reactive control of intrusions.

Overall, the validity of our model should be further investigated with high-density EEG, MEG, and direct electrophysiological recordings in the involved regions. EEG and fMRI recordings might display spurious correlations introduced by head movements (Fellner et al., 2016), although control analyses indicate that our effects are not because of movement. EEG motion artifacts were spatially filtered out with beamforming when using source activities as parametric modulators. Additionally, to optimize reconstruction accuracy of sources in the hippocampus, we followed methodological recommendations (Ruzich et al., 2019).

In summary, this study provides evidence that theta mechanisms in dACC are key to triggering inhibitory control by rDLPFC during motivated forgetting. These mechanisms can be engaged rapidly by external warning stimuli, helping to rapidly preempt unwanted thoughts. Additionally, they are strongly activated during a later time window after hippocampal retrieval likely has occurred, consistent with a reactive control response to intrusions that enhances hippocampal downregulation by the rDLPFC. This impact of PFC on hippocampal activity is achieved by rDLPFC–hippocampal beta interactions critical to clearing the mind from unwanted thoughts and to hastening the demise of memories we would prefer not to have.
